# High-fat diet-driven gut microbial sphingolipid metabolic reprogramming is associated with stress susceptibility in CUMS rats

**DOI:** 10.3389/fmicb.2026.1802003

**Published:** 2026-04-01

**Authors:** Junli Xian, Yan Li, Zhitao Feng, Yu Jin, Ting Cai, Meiqun Cao, Yongkai Cao

**Affiliations:** 1 Guangxi University of Chinese Medicine, Nanning, Guangxi, China; 2 Department of Neurology, Shenzhen Institute of Geriatrics, The First Affiliated Hospital of Shenzhen University, Shenzhen, Guangdong, China; 3 Department of Integrated Chinese and Western Medicine, Shenzhen Institute of Geriatrics, The First Affiliated Hospital of Shenzhen University, Shenzhen, Guangdong, China; 4 College of Medicine and Health Science of China Three Gorges University, Yichang, Hubei, China; 5 Department of Acupuncture and Moxibustion, The First Affiliated Hospital of Shenzhen University, Shenzhen, China; 6 Shenzhen Hospital (Futian) of Guangzhou University of Chinese Medicine, Shenzhen, Guangdong, China

**Keywords:** depression, glycerophospholipid metabolism, gut microbiota, high-fat diet, metabolites, sphingolipid metabolism

## Abstract

The escalating comorbidity between depression and metabolic syndromes induced by a high-fat diet (HFD) poses a substantial social and economic burden on society. However, the precise molecular mechanisms by which a HFD qualitatively alters the basal pathophysiology of chronic unpredictable mild stress (CUMS) remain unclear. In this study, the differential roles of microbial and metabolic pathways in the onset and exacerbation of depression were investigated using CUMS rat models fed a normal diet (ND-CUMS) or HFD (HFD-CUMS). Our findings indicated that HFD intervention showed a trend toward aggravating depressive behaviors and resulted in significantly more severe neuronal injury in the hippocampus relative to the ND-CUMS group. Notably, integrated multi-omics (metagenome and metabolome) analysis revealed a crucial pathway divergence: basal CUMS depression was strongly associated with the dysregulation of glycerophospholipid metabolism, linked to microbiota such as *Bacteroides thetaiotaomicron* and *Terrisporobacter glycolicus*, while HFD triggered a predominant disruption of the sphingolipid metabolism pathway. Exploratory mediation analysis suggested that a sphingolipid-related signature that may statistically connect HFD-associated microbial shifts with neural injury and behavioral readouts. Therefore, our findings reveal a distinct mechanistic shift underpinning metabolic-comorbid depression. HFD does not merely exacerbate stress-induced depression but fundamentally transitions the underlying pathology from glycerophospholipid to sphingolipid signaling, highlighting the potential of targeting specific lipid metabolic reprogramming as a promising therapeutic strategy for combating metabolic-comorbid depression.

## 1 Introduction

Depression is a common mental disorder primarily characterized by persistent low mood, anhedonia, and functional impairment (Rakel, 1999; McCarron et al., 2021), affecting over 280 million individuals globally, accounting for approximately 6% of the global disability burden (McCarron et al., 2021). Furthermore, the prevalence is generally higher in women than in men, imposing a substantial social and economic burden (Monroe and Harkness, 2022). Although significant progress has been made in understanding the pathophysiology of depression, including HPA axis dysfunction, monoamine deficits, and neuroinflammation, the precise etiology of depression remains elusive. However, no single hypothesis can fully explain the disorder’s complexity and high rate of treatment resistance (Rakel, 1999; Nestler and Hyman, 2010; Cui et al., 2024).

In recent years, the gut-brain axis has been associated with depression (Simpson et al., 2021; Zhang et al., 2025). Research indicated that stress exposure can induce gut microbiota dysbiosis, thereby increasing gut permeability and triggering immune responses that compromise both the gut barrier and the blood–brain barrier (Dudek et al., 2020). This allows harmful molecules and immune cells to infiltrate brain tissue, triggering oxidative stress, ultimately leading to synaptic damage and cognitive decline (Misiak et al., 2020; Loh et al., 2024). This bidirectional crosstalk links the intestinal microbiota, gut wall integrity, and the central nervous system (CNS) signaling (Patel et al., 2025). The gut microbiota, therefore, acts as a key regulator within this axis, influencing the HPA axis and brain function through systemic and peripheral pathways (Góralczyk-Bińkowska et al., 2022). Beyond its role in maintaining intestinal barrier integrity, the gut microbiota serves as a primary endogenous source of bioactive lipids. Specifically, the biosynthetic repertoire of several commensal taxa, most notably within phylum *Bacteroidetes* (e.g., *B. thetaiotaomicron*), possess the specialized enzymatic machinery required to initiate *de novo* sphingolipid synthesis (Brown et al., 2019; Wang et al., 2024a). These microbially-derived sphingolipids can translocate across the intestinal epithelium and enter host systemic circulation, thereby functioning as distal signaling molecules that shape central lipid homeostasis via the microbiota-sphingolipid-brain axis.

Emerging evidence underscores lipid homeostasis as a fundamental neurobiological interface linking metabolic status to depressive phenotypes. In Major Depressive Disorder (MDD), lipid biology extends beyond providing structural scaffolding for neuronal membranes and myelin. Lipids are fundamental determinants of membrane fluidity and lipid raft stability, which collectively govern the spatial localization and signaling transduction efficiency of key receptors and transporters (Gulbins et al., 2013). Specifically, perturbations in mitochondrial phospholipids can compromise membrane integrity and oxidative phosphorylation, thereby increasing neuronal vulnerability (Paradies et al., 2018; Bernal-Vega et al., 2023). While the hydrolysis of sphingomyelin into long-chain ceramides by acid sphingomyelinase acts as a pivotal ‘molecular switch’ that triggers ceramide-mediated apoptosis and neuroinflammation. Consequently, these mechanisms provide a robust rationale for focusing on glycerophospholipid and sphingolipid remodeling as core neurobiological layers connecting chronic stress, dietary factors, and depressive phenotypes.

Many studies have established a vicious cycle between depression and obesity; specifically, individuals with obesity are at twice the risk of depression (Plackett, 2022). High-fat diets (HFD) are a primary driver of obesity and are closely associated with changes in gut microbiota (Schoeler and Caesar, 2019; Teeple et al., 2023). In this regard, the above-mentioned gut-brain axis is critical when considering comorbidities, particularly the strong bidirectional link between depression and obesity.

While the comorbidity of depression and obesity is well-acknowledged, the precise molecular mechanisms by which high-fat diets (HFD) exacerbate depressive pathology remain poorly defined. In this study, we established a rat depression model using CUMS superimposed with an HFD intervention to simulate this comorbidity. By integrating behavioral assessments, metagenomic sequencing, and serum metabolomics, we systematically explored to distinguish the basal effect of stress from the aggravated pathology driven by diet-induced microbial and metabolic reprogramming.

## 2 Methods

### 2.1 Animals

Specific pathogen-free male Sprague–Dawley rats (150–200 g) were purchased from the Experimental Animal Center of China Three Gorges University (Yichang, China). The animal certificate number is SCXK(E) 2017–0012, and the laboratory animal use license number is SCXK(E) 2017–0061. Animals were housed under controlled conditions (12-h light/dark cycle, 20 to 26 °C, and 40 to 70% humidity) with free access to sterile water and food. After 1 week of adaptive feeding, all rats were randomly assigned to three groups: control group with normal diet (CTL, *n* = 10), depression group with normal diet (ND-CUMS, *n* = 10), depression group with high-fat diet group (HFD-CUMS, *n* = 10). To induce depressive-like behaviors, the ND-CUMS and HFD-CUMS groups were also exposed to the prescribed chronic stress regimen. All animal experimental procedures were conducted according to the Animal Welfare Guidelines and approved by the Animal Ethics Committee of the Animal Experiment Center of China Three Gorges University. (Approval No.00286497).

### 2.2 Chronic unpredictable mild stress (CUMS) procedure

The CUMS depression model was established following previously described protocols (Liu et al., 2018; Antoniuk et al., 2019; Bu et al., 2022). The stress regimen included water deprivation (24 h), food deprivation (24 h), tail clamping (2 min, 5 cm from the tip), cold water swimming (4 °C, 5 min), circadian reversal (12 h light/12 h dark), cage tilting (45° for 24 h), and damp bedding (18 h). These stressors were randomly administered over 46 days, ensuring no single stressor was repeated within 3 days to prevent anticipation. The CTL group was maintained under standard conditions.

### 2.3 Behavioral evaluation

The spontaneous activity and exploratory ability of rats were assessed using the Open Field Test (OFT) and the Elevated Plus Maze (EPM). Behavior was recorded and analyzed using a video-tracking system (TopScanLife software).

1. Open field test (OFT): Spontaneous locomotion and exploratory activity were assessed 24 h after termination of the chronic-stress paradigm. Rats were gently handled in the testing room for 5 min/d on 3 consecutive days before behavioral assessment. The open-field apparatus was a gray PVC box (44 × 44 × 47 cm) with a non-transparent floor and walls. At the start of each session, an individual rat was placed gently in the center of the arena, and its behavior was recorded for 5 min with a computer-based video-tracking system (ENV-520, Med-Associates, USA). Between animals, the floor and walls of the arena were wiped with 75% ethanol and allowed to air-dry to eliminate olfactory cues. Testing was conducted in a quiet room, and animals from different experimental groups were evaluated in an alternating manner. Behavioral parameters extracted offline included total distance traveled (cm), distance traveled within the central zone, and immobility duration (s).
2. Elevated Plus Maze (EPM): The subjects were placed in the center area (facing the open arms) and observed for 5 min using a video behavioral analysis system to record their activity within the elevated plus maze. The percentage of time spent in the open arms (OT%) was calculated as ratio of the time spent in the open arms to the total time spent in both open and closed arms. The percentage of open arms entries (OE%) was defined as the ratio of entries into the open arms to the total into both open and closed arms. This analysis was performed to evaluate the duration the subjects spent in the maze (Walf and Frye, 2007).

### 2.4 HE staining and Nissl staining of hippocampal tissue

Hippocampal tissue samples from each group were fixed, dehydrated, and embedded in paraffin; the paraffin blocks were then cut into 5 μm-thick sections. Sections were dried in a 60 °C oven for 4 h, dewaxed in xylene twice (15 min each), rehydrated in 95, 85, and 75% ethanol for 2 min each, treated with distilled water for 20 min, stained with eosin for 3 min, dehydrated with ethanol at different concentrations (75, 85, 95%), and finally observed under an optical microscope.

Portions of hippocampal tissue samples from each group were frozen-fixed in a cryostat at −20 °C; after the tissue became hardened and white, serial sections were prepared. The 10 μm cryosections were dried, incubated with Nissl staining solution for 5 min, differentiated with 950 mL/L ethanol, mounted with neutral resin, and observed under a microscope to count Nissl-positive cells.

### 2.5 Fecal metagenomic sequencing

Fecal samples (*n* = 5 per group) were collected from three experimental groups after the behavioral tests. Total genomic DNA was extracted from 200 to 300 mg of feces using mechanical lysis followed by centrifugation. The supernatant was treated with binding and wash buffers, and DNA was finally eluted using an elution buffer. A sequencing library (300–400 bp insert size) was constructed through DNA fragmentation, end repair, A-tailing, adapter ligation, and PCR amplification. The resulting libraries were circularized to form DNA nanoballs (DNBs), which were sequenced on the BGISEQ platform using combinatorial Probe-Anchor Synthesis (cPAS). Raw sequencing data underwent quality control, assembly, gene prediction, and taxonomic annotation. Taxa abundance was summarized from gene counts, and differential abundance analysis was performed, including species diversity and LEfSe.

### 2.6 Collection of serum samples and metabolomics analysis

Blood was collected from the rats and allowed to stand at room temperature to separate the serum via centrifugation. A 50 μL serum sample was mixed with 200 μL of cold methanol (containing an internal standard) and vortexed. The mixture was then centrifuged at 14,000 *g* for 15 min at 4 °C. The supernatant (200 μL) was transferred to a −20 °C freezer for storage. Before analysis, the concentrated extracts were re-dissolved in 100 μL of 20% methanol/water solution until fully dissolved, followed by vortexing and centrifugation to collect the supernatant for positive and negative ion mode analysis.

All measurement data were collected using Analyst® TF software. Primary mzmL files, secondary mgf files, peak tables, and peak detection were extracted using OneMap-PTO software, with normalization parameters and retaining 50 secondary mass spectrometry fragments. Qualitative analysis of the detected peak tables was carried out using the One-Map database. Multivariate data were imported into the SIMCA software package for discriminative model fitting analysis to obtain information on variables contributing to classification. The stability and predictive capability of the OPLS-DA model were assessed using R2X and Q2 to determine the effectiveness of the model fit. Additionally, a *t*-test and orthogonal partial least squares discriminant analysis (OPLS-DA) were conducted to identify key differential metabolites (*p* < 0.05, Variable Importance in Projection [VIP] > 1) using the MetaboAnalyst database.

### 2.7 Integrated correlation analysis of gut microbiota, serum metabolites, and behavioral phenotypes

Based on the two overlapping differential features: the overlapping differential microbes and metabolites between the (ND-CUMS vs. CTL) and (HFD-CUMS vs. CTL) datasets, and the overlapping differential features from the (ND-CUMS vs. HFD-CUMS) and (HFD-CUMS vs. CTL) datasets, we performed a comprehensive integration analysis to delineate the functional link between the gut microbiome, host metabolism, and behavior.

Spearman correlation analysis was applied to identify the significant associations between microbial taxa and specific metabolites. The top 50 strongest correlations were visualized using heatmaps.

The metabolic origin of differential metabolites from host, microbial, or co-metabolism was matched and classified by MetOrigin 2.0 (Yu et al., 2024). Subsequently, metabolic pathway enrichment was implemented separately for metabolites from different sources. The biological association between microbiota and metabolite was identified via a systematic search of bacterial species that may participate in the metabolism. Integrating the statistical correlation, the resulting high-confidence, multi-level interaction structure, connecting microbial taxa (from phylum to species) to metabolites, was then visualized using a Sankey diagram.

The mediation analysis was then employed to comprehensively delineate the hypothetical mediation pathways between microbiota and phenotypes mediated by metabolites. Specifically, spearman correlation was performed on metabolites with bacteria and phenotypes, respectively. The metabolites that exhibit significant correlations with both microbiota and phenotypes were identified and visualized through a Sankey diagram together with related bacteria and phenotypes.

### 2.8 Statistical analysis

All behavior data were analyzed and visualized by GraphPad Prism V9.5. Other statistical analyses were performed using R V4.5. Student’s t-test was used to compare metabolite abundances between groups. Non-parametric Wilcoxon rank-sum tests were applied to assess differences in microbial species abundance between groups. Spearman’s rank correlation was accomplished using the psych R package. Origin analysis and pathway enrichment analysis were performed by MetOrigin 2.0 and visualized by the plotly package. Mediation pathways were calculated by an R script using the plotly package and visualized by Cytoscape. For mediation modeling, we estimated ACME (indirect effect) and ADE (direct effect) using nonparametric bootstrapping. We further assessed robustness via sensitivity resampling, summarized by direction-consistency rates (e.g., stability dir rate and stability same sign rate). Boxplots were generated using the ggplot2 R package. The significance threshold for all statistical tests was set at *p* < 0.05.

## 3 Results

### 3.1 Effect of HFD on the behavior of CUMS rats

**Figure 1:**
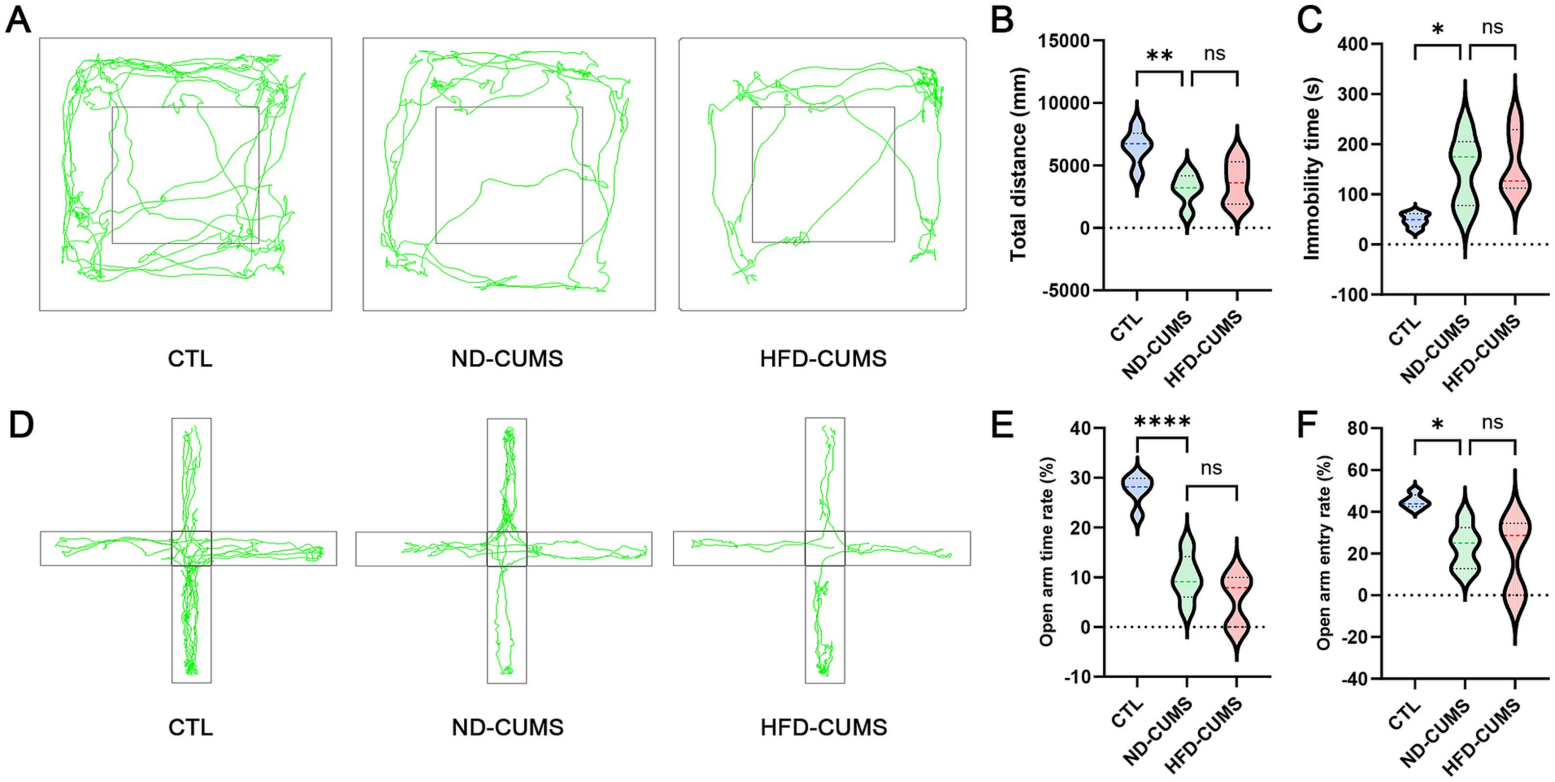
Effects of CUMS under different dietary backgrounds on locomotor activity and anxiety-like behavior in rats. **(A)** Representative movement trajectories in the open field test (OFT). **(B)** Total distance traveled in the OFT. **(C)** Immobility time in the OFT. **(D)** Representative trajectories in the elevated plus maze (EPM). **(E)** Percentage of time spent in the open arms in the EPM. **(F)** Open-arm entry rate in the EPM. Data are presented using violin plots combined with internal quartile representations. Compared with the CTL group, *p < 0.05; **p < 0.01 and ****p < 0.0001; ns, not significant.

We examined the effect of HFD on anxiety-like and depression-like behaviors induced by CUMS in rats. In the Open Field Test (OFT), representative movement trajectories (Figure 1A) visually demonstrated restricted exploratory behavior in both the ND-CUMS and HFD-CUMS groups compared to the Control (CTL) group. Intriguingly, the movement trajectory of the HFD-CUMS group was further shortened compared with the ND-CUMS group. Quantitative analysis confirmed that the total distance traveled was significantly reduced in both CUMS groups (Figure 1B, *P* < 0.01). Consistently, the immobility time in the ND-CUMS and HFD-CUMS groups was significantly increased compared to the CTL group (*p* < 0.05) (Figure 1C). While the behavioral differences between HFD-CUMS and ND-CUMS groups did not reach statistical significance in the OFT, the observed trends in total distance and immobility time suggest a potential latent burden imposed by HFD, which may predispose rats to more severe psychomotor retardation under chronic stress.

In the Elevated Plus Maze (EPM) test, representative trajectories (Figure 1D) showed reduced exploration of open spaces in the CUMS groups. Compared with the CTL group, rats in both CUMS groups exhibited a significantly reduced percentage of time spent in the open arms (Figure 1E, *P* < 0.05) and lower open-arm entry rates (Figure 1F, *P* < 0.05). The HFD-CUMS group showed a non-significant, but consistently worsening trend compared to the ND-CUMS group.

### 3.2 Effect of HFD on the pathology of CUMS rats

**Figure 2:**
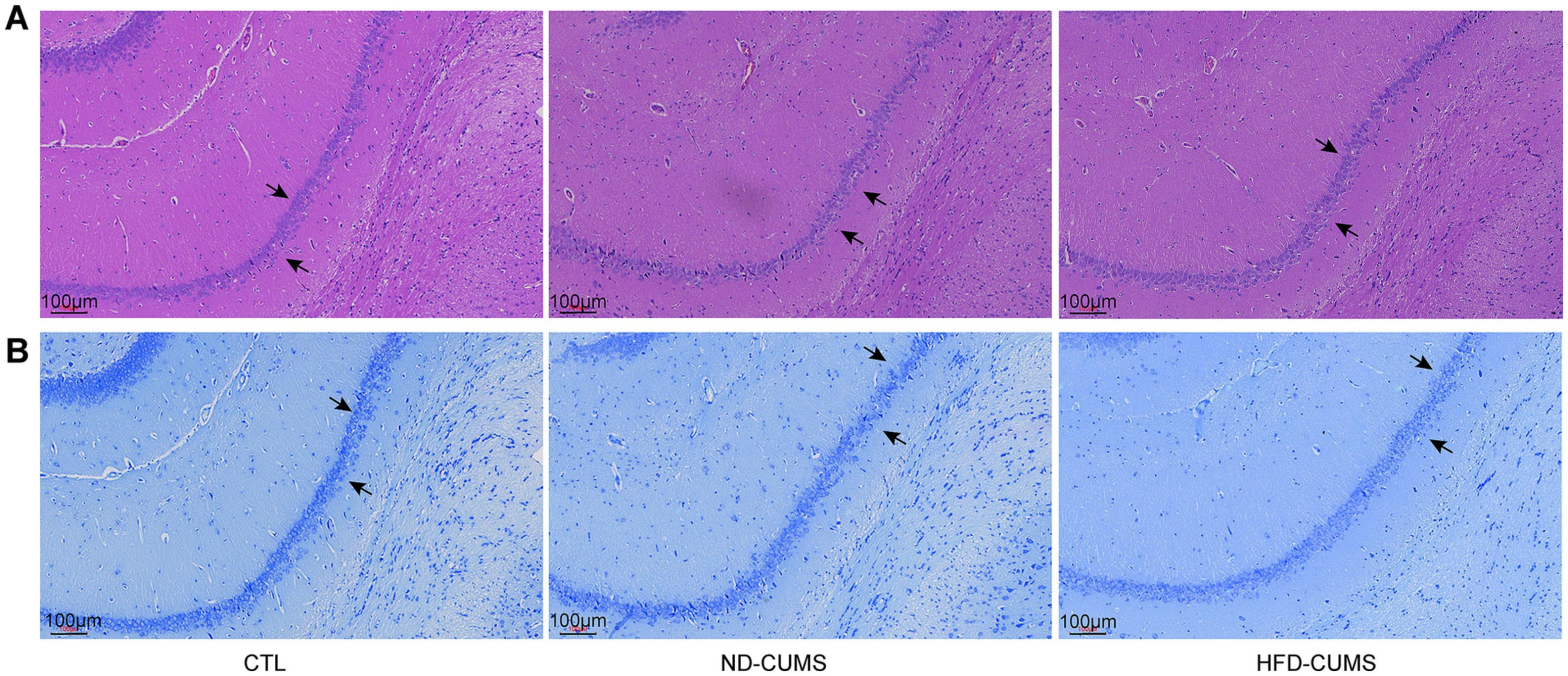
Histopathological evaluation of the hippocampal CA1 region in the rat. **(A)** Representative Hematoxylin and Eosin (HE) staining images (magnification, ×100) and quantitative analysis of the number of neurons. Black arrows indicate damaged neurons characterized by irregular arrangement, shrunken cell bodies. **(B)** Representative Nissl staining images (magnification, ×100). Black arrows indicate a reduction in the density of Nissl bodies.

Histopathological evaluations assessed neuronal integrity in the hippocampal CA1 region (Figure 2). Hematoxylin and Eosin (H&E) staining (Figure 2A) assessed neuronal morphology in the hippocampal CA1 region. CTL rats showed densely packed and well-organized neurons with prominent nucleoli. In contrast, the ND-CUMS group displayed clear neuronal damage, characterized by widened intercellular spaces, irregular arrangement, and shrunken cells with pyknotic nuclei. These pathological alterations were markedly exacerbated in the HFD-CUMS group, which showed severe cellular disorganization and extensive nuclear loss.

Nissl staining (Figure 2B) provided further evidence of neuronal degeneration. CTL neurons were rich in Nissl bodies (rough endoplasmic reticulum), reflecting high metabolic activity. Conversely, the ND-CUMS group exhibited a notable reduction in Nissl density. This degeneration was most pronounced in the HFD-CUMS group, where neurons displayed dissolution of Nissl bodies. Collectively, these findings support that the HFD synergistically amplifies the neurodegeneration induced by chronic stress.

Interestingly, while the HFD-CUMS group did not exhibit a statistically significant exacerbation of behavioral deficits compared to the ND-CUMS group (as shown in Figure 1), histological analysis revealed a markedly different pattern. As shown in Figure 2, HFD-CUMS rats displayed significantly more severe neuronal disorganization and degeneration, indicating a clear exacerbation of neuropathology despite similar behavioral outputs.

### 3.3 Effect of HFD on the gut microbiota of CUMS rats

**Figure 3:**
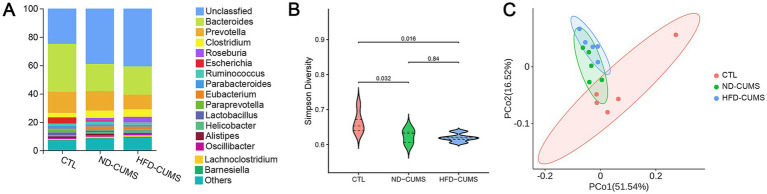
Effects of CUMS under different dietary backgrounds on gut microbiota composition and diversity. **(A)** Genus-level stacked bar plots of relative abundances in CTL, ND-CUMS, and HFD-CUMS groups. **(B)** Violin plots of the Simpson diversity index (*α* diversity). **(C)** PCoA scatter plot based on the community distance matrix.

We evaluated changes in the gut microbiome using metagenomic sequencing. The total number of OTUs detected at the phylum, class, order, family, genus, and species levels was 59, 84, 165, 355, 1283, and 4966, respectively. At the genus level, the community was primarily composed of *Bacteroides*, *Prevotella*, *Clostridium*, *Roseburia*, *Escherichia*, and *Ruminococcus* (Figure 3A, Supplementary table 1). The community diversity (*α* diversity) presented by the Simpson index indicated that CUMS exposure significantly reduced the diversity of gut microbes. Specifically, both the ND-CUMS group (*p* = 0.032) and the HFD-CUMS group (*p* = 0.016) exhibited significantly lower diversity indices compared to the CTL group. Notably, no significant differences were observed between the ND-CUMS and HFD-CUMS groups (*p* = 0.84), suggesting that HFD intervention does not further reduce microbial diversity but rather drives a qualitative reprogramming of the gut microbiota (Figure 3B). The Principal Coordinate Analysis (PCoA) of the bacterial community (*β*-diversity) showed that PCo1 and PCo2 explained 51.54 and 16.52% of the total variation, respectively (Figure 3C). The CTL group formed a distinct cluster, well-separated from the two CUMS groups. While the ND-CUMS and HFD-CUMS groups showed some overlap, they exhibited a discernible spatial divergence. This indicated that while HFD and ND sharing a common stress-induced microbial background, HFD drives a specific qualitative reprogramming of the community composition that distinguishes it from the ND-CUMS profile, rather than merely causing progressive dysbiosis.

**Figure 4:**
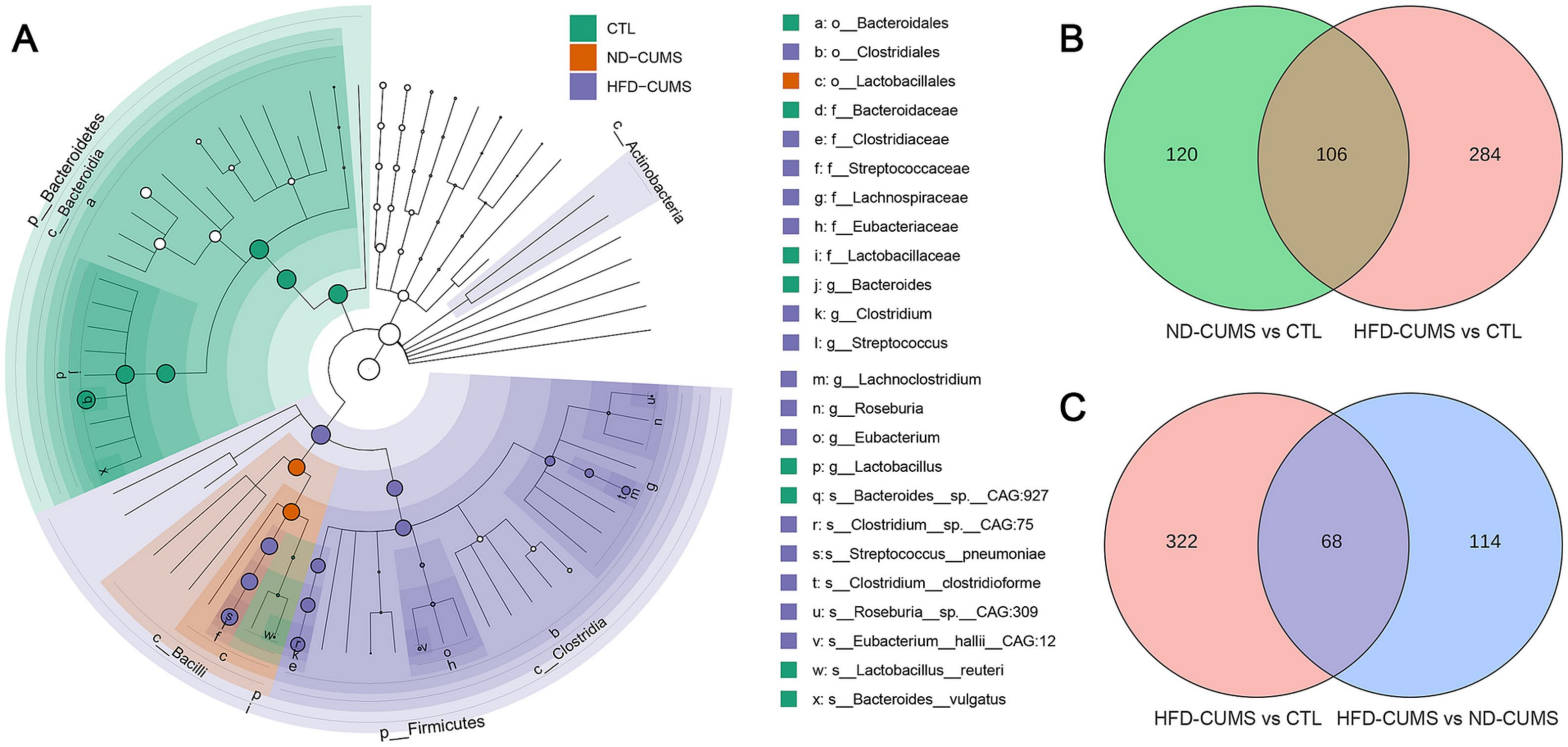
Effects of CUMS under different dietary backgrounds on characteristic bacterial taxa in the gut microbiota in rats. **(A)** LEfSe phylogenetic trees. **(B)** Venn diagram of differential taxa between ND-CUMS vs. CTL and HFD-CUMS vs. CTL. **(C)** Venn diagram of differential taxa between HFD-CUMS vs. CTL and HFD-CUMS vs. ND-CUMS.

To identify characteristic bacterial taxa, we constructed a linear discriminant analysis effect size (LEfSe) analysis (Figure 4A, Supplementary table 2). At the order level, the *Bacteroidales* were the most significant characteristics of the CTL group, while the *Lactobacillales* characterized the ND-CUMS group. Key marker bacteria in the CTL group included the g_*Bacteroides*, such as s_*Bacteroides* sp. CAG:927, s_*Bacteroides vulgatus*, and s_*Lactobacillus reuteri*. In contrast, the HFD-CUMS treatment significantly altered the microbial community structure, enriching marker genera such as g_*Clostridium*, g_*Streptococcus*, g_*Lachnoclostridium*, g_*Roseburia*, and g_*Eubacterium*. Notably, at the species level, multiple potentially pathogenic or conditionally pathogenic species are enriched in the HFD-CUMS group, including s_*Streptococcus pneumoniae* and s_*Clostridium clostridioform*.

The Venn diagrams were constructed to identify overlapping differential species (Figures 4B,C). Using *p* < 0.05 and FC > 2 thresholds, the intersection between “ND-CUMS vs. CTL” and “HFD-CUMS vs. CTL” revealed 106 shared differential species, with 120 and 284 unique species, respectively (Figure 4B). The overlap between “HFD-CUMS vs. CTL” and “HFD-CUMS vs. ND-CUMS” included 68 shared species, with 322 and 114 unique species, respectively (Figure 4C).

### 3.4 Effect of HFD on the serum metabolism of CUMS rats

**Figure 5:**
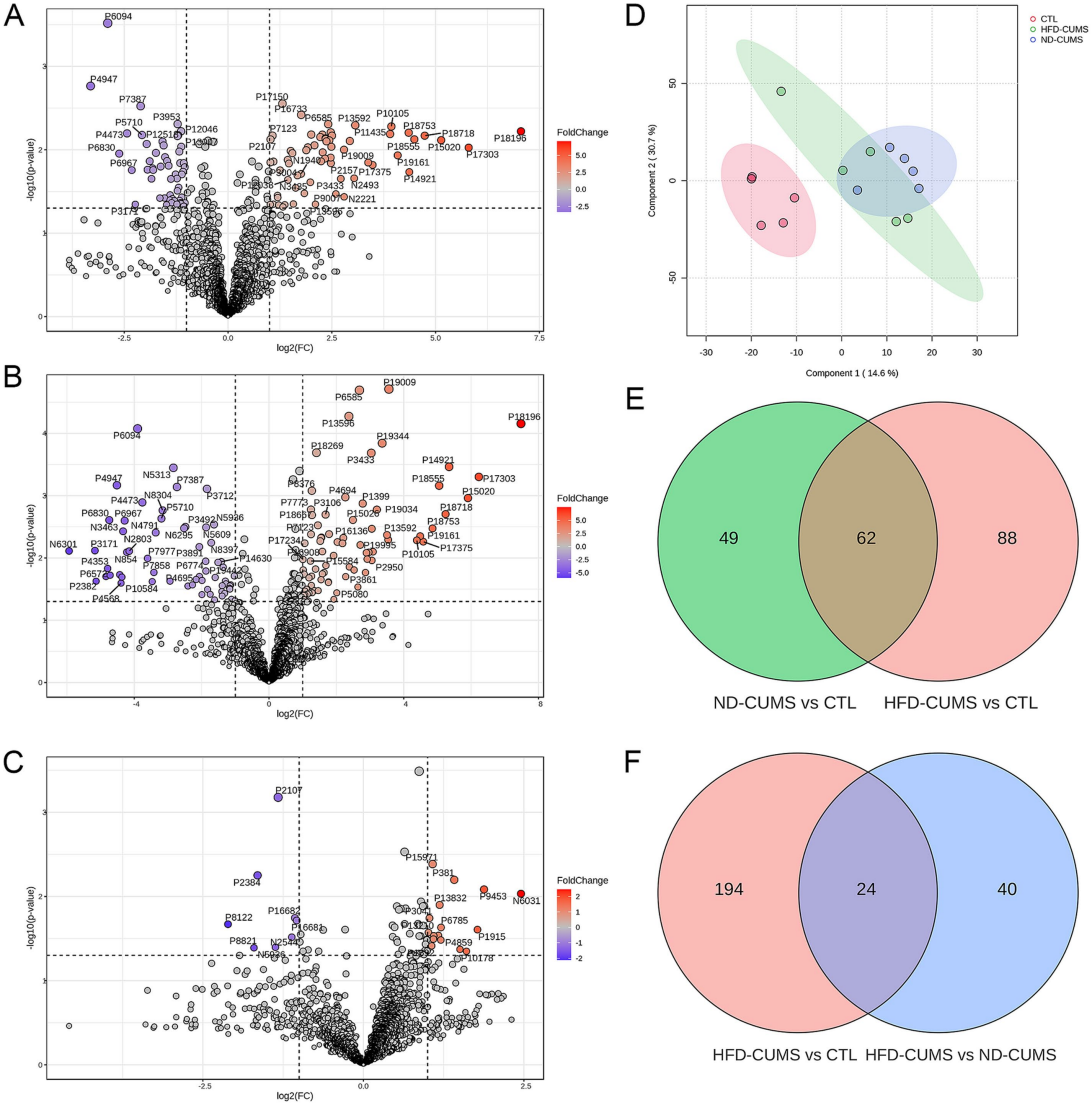
Effects of CUMS under different dietary backgrounds on serum metabolic profiles in rats. **(A–C)** Volcano plots showing differentially expressed metabolites: ND-CUMS vs. CTL, HFD-CUMS vs. CTL, and HFD-CUMS vs. ND-CUMS, respectively. **(D)** PLS-DA score plot among CTL, ND-CUMS, and HFD-CUMS groups. **(E)** Venn diagram between ND-CUMS vs. CTL and HFD-CUMS vs. CTL. **(F)** Venn diagram between HFD-CUMS vs. CTL and HFD-CUMS vs. ND-CUMS.

Differential metabolites were identified using specific screening criteria, as visualized in the volcano plots (Figures 5A–C, Supplementary table 3 and 4). Initially, under a strict screening criterion (*p* < 0.05 and FC > 2), 78 up-regulated and 38 down-regulated metabolites were identified in the ND-CUMS compared to the CTL group. Similarly, the HFD-CUMS vs. CTL comparison yielded 74 up-regulated and 76 down-regulated metabolites. To capture broader metabolic reprogramming between the stress and diet models, a less stringent threshold (*p* < 0.05 and FC > 1.5) was applied, which revealed 113 up-regulated and 105 down-regulated metabolites in the HFD-CUMS vs. CTL group, and 35 up-regulated and 29 down-regulated metabolites in the HFD-CUMS vs. ND-CUMS. The score plot of the PLS-DA model revealed the distinct serum metabolic profiles among the three groups, indicating significant metabolic separation based on stress and diet interventions (Figure 5D).

Venn diagrams illustrated the overlaps of these differential features: in the comparisons against the CTL group (FC > 2), 62 metabolites were common to both ND-CUMS and HFD-CUMS groups, with 49 and 88 unique metabolites, respectively (Figure 5E). Furthermore, when comparing the differential metabolites of HFD-CUMS vs. CTL and HFD-CUMS vs. ND-CUMS (FC > 1.5), 24 metabolites were found to be common between these contrasts (Figure 5F).

### 3.5 CUMS-induced depression correlates with glycerophospholipid metabolism

**Figure 6:**
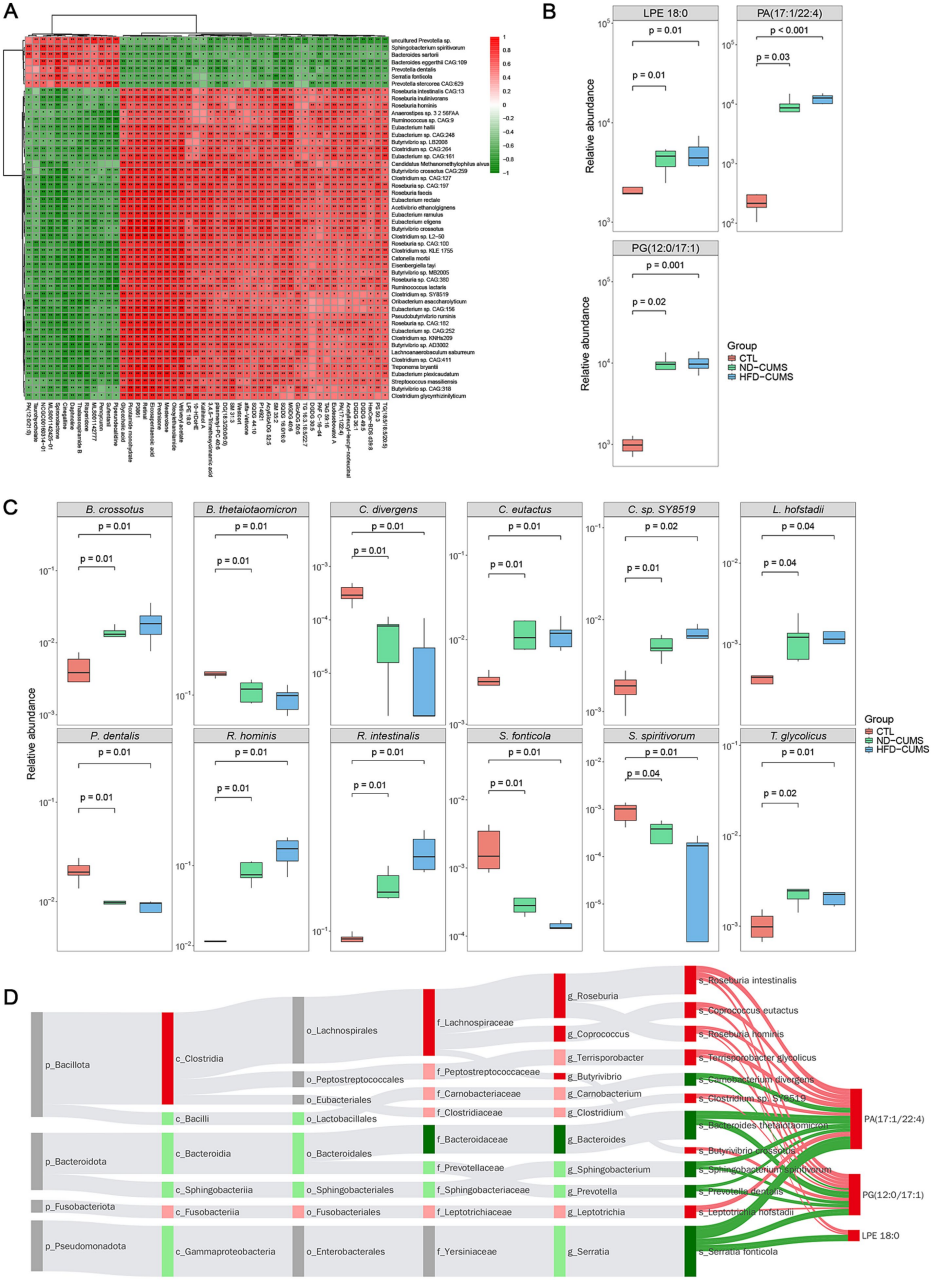
Correlation analysis between shared differential gut microbiota and serum metabolites in the [(ND-CUMS vs. CTL) ∩ (HFD-CUMS vs. CTL)] group. **(A)** Heatmap showing Spearman correlations between shared differential metabolites and key bacteria; red indicates positive correlations, green indicates negative correlations, and color intensity reflects the correlation coefficient. **(B)** Relative abundances of typical correlated metabolites in the three groups of rats. **(C)** Box plots comparing the relative abundances of bacteria are highly correlated with shared metabolites among CTL, ND-CUMS, and HFD-CUMS groups. **(D)** Sankey diagram illustrating the potential metabolic pathways linking shared metabolites with significantly associated bacterial taxa, displayed hierarchically from phylum (p_) to species (s_) level; the width of the connecting lines represents the strength of the correlation.

We employed the Spearman correlation analysis to investigate the relationships between the core overlapping differential gut microbiota and metabolites related to depression (Figure 6A). Significant correlations were identified between several key differential bacteria and metabolites. For instance, bacteria such as *Roseburia hominis* and *Butyrivibrio crossotus* exhibited positive correlations with the lipids PA (17:1/22:4) and LPE 18:0 (*P* < 0.05 or *P* < 0.01). Conversely, *Sphingobacterium spiritivorum*, *Serratia fonticola*, and *Prevotella dentalis* showed significant negative correlations with the same metabolites (*P* < 0.01).

Metabolic origin analysis and subsequent metabolic pathway enrichment demonstrated that the shared differential metabolites between (ND-CUMS vs. CTL) and (HFD-CUMS vs. ND-CUMS) were primarily enriched in the glycerophospholipid metabolism pathway (Supplementary Figure 1). A high-confidence interaction network was constructed, linking the three shared glycerophospholipids with 12 significantly associated microbial species (Figure 6D). The identified metabolites were LPE 18:0, PA (17:1/22:4), and PG (12:0/17:1), and the species included *Bacteroides thetaiotaomicron*, *Terrisporobacter glycolicus*, *Roseburia intestinalis*, and *Coprococcus eutactus*.

As shown in Figure 6C, both CUMS treatments significantly altered the abundance of multiple gut microbiota compared to the CTL group. The abundances of *Butyrivibrio crossotus*, *Coprococcus eutactus*, *Clostridium* sp. SY8519, *Leptotrichia hofstadii*, *Roseburia hominis*, and *Roseburia intestinalis* were significantly increased in both CUMS groups. Conversely, the abundances of *Bacteroides thetaiotaomicron*, *Carnobacterium divergens*, *Prevotella dentalis*, *Serratia fonticola*, and *Sphingobacterium spiritivorum* were significantly decreased. Notably, the abundance of *Terrisporobacter glycolicus* was significantly increased in both CUMS groups. Metabolomic analysis revealed that CUMS exposure significantly elevated the levels of specific metabolites, regardless of diet (Figure 6B). Specifically, the concentrations of LPE 18:0, PA (17:1/22:4), and PG (12:0/17:1) were significantly higher in both the ND-CUMS and HFD-CUMS groups compared to the CTL group. This indicates that the upregulation of these glycerophospholipids is a hallmark of chronic stress exposure, not dietary fat intake.

Finally, exploratory mediation analysis (utilizing non-parametric bootstrap resampling) was conducted to assess whether glycerophospholipid variations could potentially link microbial shifts to behavioral readouts. While the models suggested some directional trends, the majority of estimated indirect (ACME) and direct effects (ADE) yielded 95% confidence intervals that overlapped zero, and stability assessments revealed insufficient robustness for most pathways (Supplementary table 5). Consequently, rather than establishing a definitive causal chain, these findings are considered hypothesis-generating; the detailed mediation parameters and sensitivity analysis results are provided in the Supplementary Material (Supplementary Figure 3). Despite the statistical limitations of the mediation models, the qualitative alignment between specific microbial shifts and metabolic alterations remains a notable feature of our two-axis model, providing a basis for understanding the distinct metabolic trajectories under different dietary conditions.

### 3.6 HFD-driven stress susceptibility is linked to sphingolipid metabolism

**Figure 7:**
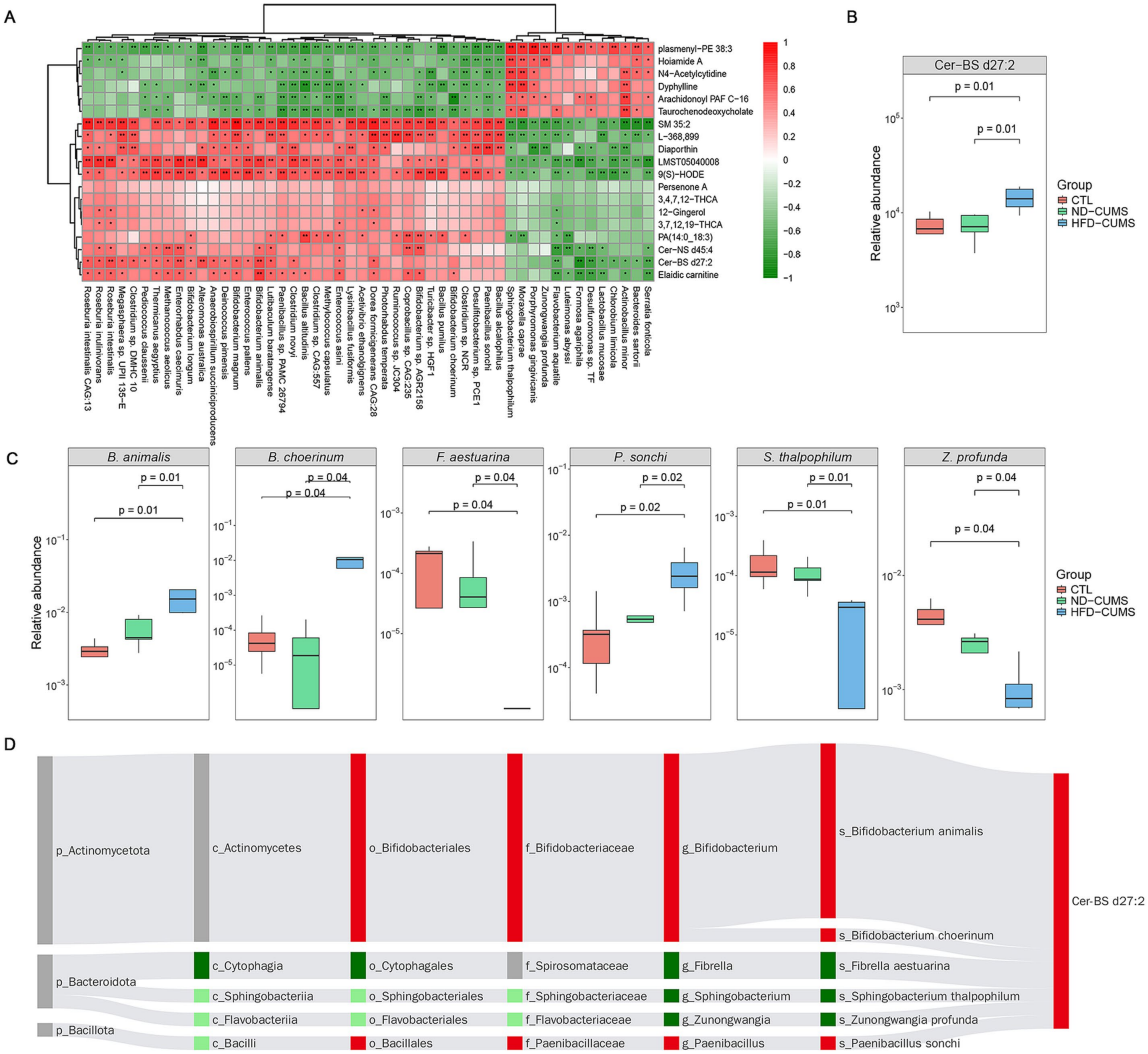
Association analysis between differentially abundant microbial communities and serum metabolites in the group [(HFD-CUMS vs. ND-CUMS) ∩ (HFD-CUMS vs. CTL)]. **(A)** Heatmap showing Spearman correlations between shared differential metabolites and key bacteria; red indicates positive correlations, green indicates negative correlations, and color intensity reflects the correlation coefficient. **(B)** Box plots compare the level differences of Cer-BS d27:2 among the CTL, ND-CUMS, and HFD-CUMS groups. **(C)** Relative abundances of typical correlated gut microbiota in the three groups of rats. **(D)** Sankey diagram illustrating the potential metabolic pathways linking shared metabolites with significantly associated bacterial taxa, displayed hierarchically from phylum (p_) to species (s_) level; the width of the connecting lines represents the strength of the correlation.

To identify the specific mechanism driven by the HFD, we focused on the overlapping features unique to the HFD model (identified in Figures 4C, 5F) and applied Spearman correlation analysis. As shown in Figure 7A, *Bifidobacterium animalis* showed a positive correlation with Cer-BS d27:2 (*p* < 0.01).

Integrated metabolic pathway enrichment revealed that the differential microbiota and metabolites driven by HFD were primarily enriched in the sphingolipid metabolism pathway (Supplementary Figure 2). The resulting interaction network (Figure 7D) specifically links the key sphingolipid metabolite Cer-BS d27:2 to several microorganisms, including *Bifidobacterium animalis, B. choerinum, Fibrella aestuarina, Sphingobacterium thalpophilum, Zunongwangia profunda, and Paenibacillus sonchi*. The Sankey diagram maps these connections from the phylum to the species level, with the thickness of the lines indicating the strength of the correlation.

The differential analysis revealed that Cer-BS d27:2 levels in the HFD-CUMS group were significantly elevated (*p* < 0.01) and exceeded those in the ND-CUMS group or control group by approximately two-fold (Figure 7B). Crucially, the distinct microbial reprogramming, including increased *Bifidobacterium animalis*, *B. choerinum*, and *Paenibacillus sonchi*, alongside decreased *Fibrella aestuarina*, *Sphingobacterium thalpophilum*, and *Zunongwangia profunda*, was driven primarily by HFD but not by stress, as these features show no significant deviations between ND-CUMS and CTL groups (Figure 7C).

Similar to the observations in the glycerophospholipid axis, exploratory mediation analyses for the sphingolipid-mediated pathways also revealed a lack of statistical robustness, with most 95% confidence intervals overlapping zero (Supplementary table 6). Consequently, these mediation parameters are provided as hypothesis-generating data in the Supplementary Material (Supplementary Figure 4). Despite this, the qualitative alignment between HFD-enriched species (e.g., *Bifidobacterium animalis*) and the upregulation of Cer-BS d27:2 remains a key characteristic of the HFD-driven metabolic shift.

## 4 Discussion

In recent years, the central role of dietary factors in the pathogenesis and exacerbation of depression has become increasingly prominent, with the HFD confirmed as a key influencing factor in the pathophysiology of this mental disorder (Asimakidou et al., 2025). Additionally, HFD-induced metabolic dysregulation, including obesity, insulin resistance, and metabolic syndrome, establishes a critical bidirectional relationship with mental health issues, further emphasizing the complex interplay between nutrition and mental health (GBD 2021 Risk Factors Collaborators, 2024). The understanding of these dietary influences on depression is crucial in developing effective prevention.

Our study first investigated the combined impact of HFD and CUMS on depression-like behaviors. Consistent with the CUMS model, rats exposed to stress, regardless of their diet, exhibited core depressive-like phenotypes: a significant reduction in total distance and exploratory desire (open arm entries), and an increase in immobility time in EPM and OFT. The HFD-CUMS displayed a consistent, but statistically non-significant, exacerbation trend in the neurobehavioral manifestations compared with ND-CUMS. Notably, the mismatch between non-significant behavioral changes and significant histological damage suggests that HFD acts as a metabolic amplifier of vulnerability rather than a primary behavioral driver in the short term. These findings indicate that HFD-induced metabolic reprogramming may represent a “latent vulnerability” that precedes overt behavioral divergence. The lack of statistical significance in exacerbated behavior between the HFD-CUMS and ND-CUMS groups may be attributed to several factors. Apart from sample size limitations or a potential ceiling effect of the CUMS model, it is plausible that a more prolonged duration of HFD intervention or the utilization of more sensitive behavioral paradigms, such as the sucrose preference test (SPT) across multiple time points, would be required to capture a statistically significant manifestation of the HFD-driven ‘latent vulnerability’. These results suggest that while neurostructural damage is significantly advanced by HFD, its full conversion into overt behavioral despair may require a longer temporal window.

However, when linking this trend with the profound underlying metabolic and microbial reprogramming, we propose that HFD is not merely an independent behavioral trigger, but rather a subclinical environmental risk factor that significantly enhances the susceptibility of the gut-brain axis to chronic stress injury. This subtle behavioral aggravation, substantiated by multi-omics data, warrants further investigation with larger sample sizes to clarify the role of HFD in intensifying depressive-like symptoms.

Our LEfSe analysis delineated distinct microbial profiles corresponding to the host’s metabolic and stress status. The CTL group was predominantly characterized by short-chain fatty acids (SCFAs)-producing bacteria, such as o_*Bacteroidales* and g_*Bacteroides*, which are crucial for maintaining gut barrier integrity and anti-inflammatory effects. In sharp contrast, the HFD-CUMS group facilitates a reprogramming toward a potential pro-inflammatory state, characterized by a significant enrichment of genera such as *Clostridium* and *Streptococcus*, including known pathobionts like *Streptococcus pneumoniae*. This pattern, marked by a depletion of SCFA producers and an enrichment of pathogens, suggests a mechanism wherein HFD exacerbates CUMS-induced dysbiosis, potentially impairing gut barrier function and amplifying systemic inflammation, which subsequently influences neurobehavioral outcomes.

The microbiota results in species-level related complex, and in some instances contradictory microbial changes that reflect a dynamic interplay between host stress, diet, and microbial function. First, bacteria such as *Bacteroides thetaiotaomicron* were significantly reduced in both the CUMS groups compared with the CTL group. Given *Bacteroides thetaiotaomicron* (*B. theta*)‘s reported roles in alleviating metabolic syndrome, hyperlipidemia, hepatic steatohepatitis, and colitis (Brown et al., 2019; Li et al., 2024a; Wang et al., 2024b), its depletion likely contributes to the metabolic and neuroinflammatory burden observed in the CUMS model. Conversely, the genus *Terrisporobacter*, specifically *Terrisporobacter glycolicus*, was significantly enriched in both CUMS groups. Known for its association with hyperlipidemia and potential pathogenicity (Guo et al., 2023), the increase in *T. glycolicus* directly links stress and diet to heightened dysmetabolism and potential barrier disruption.

A notable finding was the significant increase in several traditionally classified butyrate-producing bacteria, including *Butyrivibrio crossotus, Roseburia intestinalis*, *Coprococcus eutactus*, and *Roseburia hominis*, in both the CUMS groups. While these species are generally anti-inflammatory and linked to depression relief (Xu et al., 2021; Patra et al., 2023; Peng et al., 2023), their increased abundance in our disease models requires a more nuanced, context-dependent interpretation. We hypothesized that this increase in abundance may represent a compensatory host response, whereby the organism attempts to induce SCFA production to counteract the pervasive inflammation and metabolic stress. Alternatively, the specific strain enriched in our rat model may possess pathobiont characteristics, or their enrichment in the disease state may associate with specific, dysregulated metabolic roles other than butyrate production.

This interpretation is consistent with the literature of *Roseburia hominis* enrichment associated with liver disease and osteoporosis (Gao et al., 2024; Li et al., 2024b), suggesting that a microbe’s effect is highly dependent on the specific physiological environment and strain. The decreased abundance of *Coprococcus eutactus* is closely associated with lower levels of the dopamine metabolite DOPAC, which further elevates its status beyond a mere SCFA producer to a potential probiotic, suggesting a direct role in regulating dopamine-related mood and motor functions (Notting et al., 2023).

Additionally, several microbiota species were demonstrated significant associations with depressive-like behaviors despite a notable scarcity of existing literature linking them directly to neuropsychiatric conditions, including *Carnobacterium divergens*. In our study, the abundance of *Carnobacterium divergens* was significantly reduced in both the CUMS groups. It has been reported that *Carnobacterium divergens* MB421, isolated from green olives, is characterized as a “potential probiotic candidate” and can be used as a starter culture for fermenting probiotic foods (Saeed et al., 2023).

Furthermore, the HFD-CUMS group exhibited a significantly increased relative abundance of *Bifidobacterium animalis* and *Bifidobacterium choerinum*. Although certain *B. animalis* strains are established probiotics against obesity (Pedret et al., 2019; Uusitupa et al., 2020; Zhao et al., 2025), their proliferation in our high-fat, chronic-stress disease state may represent another manifestation of the compensatory mechanism.

It is plausible that the HFD-CUMS condition induces a specific dysbiotic environment, and the expansion of these *Bifidobacterium* species is an adaptive attempt to restore homeostasis, possibly through counteracting inflammation or reinforcing the gut barrier. Their increases underscore the complex, context-dependent role of gut microbiota in health and disease.

Our metabolic pathway enrichment of microbial and metabolites has identified glycerophospholipid metabolism dysregulation associated with CUMS-induced depression, specifically mediated by LPE 18:0, PA (17:1/22:4), and PG (12:0/17:1), while the sphingolipid metabolism disruption accounted for HFD-exacerbated depression, mediated by Cer-BS d27:2. Notably, we propose a distinct mechanistic divergence: Stress targets membrane fluidity via glycerophospholipids, while HFD targets signaling and apoptosis pathways via sphingolipids (Xu et al., 2021; Notting et al., 2023). Sphingolipids are essential for neuronal membrane integrity, signal transduction, and apoptosis regulation (Hannun and Obeid, 2008; Heaver et al., 2018), and their dysregulation is linked to various neuropsychiatric and metabolic disorders (Hannun and Obeid, 2018; van Echten-Deckert and Alam, 2018). Our findings align with this hypothesis that an HFD disrupts the gut microbiota-sphingolipid axis, where prolonged lipid overload induces qualitative microbial and metabolic reprogramming, thereby amplifying neuro-metabolic stress and emotional deficits (Raichur et al., 2014; Johnson et al., 2020). This indicates that the HFD exacerbates depression by shifting the metabolic burden from general membrane dysregulation (glycerophospholipids) to specific apoptotic signaling (sphingolipids). This divergence highlights the need for precision medicine: therapeutic strategies for comorbid depression-obesity may require specifically targeting sphingolipid metabolism and its microbial regulators, rather than standard monoamine pathways.

Our multi-omics integration provides consistent qualitative associations among gut microbial composition, lipid metabolic profiles, and hippocampal pathology, supporting a biologically plausible ‘gut–lipid–brain’ axis. Although exploratory mediation models did not yield robust statistical significance for every pathway—likely due to the inherent constraints of sample size—the qualitative consistency between the depletion of beneficial taxa (e.g., *Bacteroides thetaiotaomicron*) and the dysregulation of key neuronal lipids, including PA, PG, and LPE, is noteworthy. Mechanistically, these lipids are recognized as critical components of neuronal health (Choudhary et al., 2024; Giblin et al., 2025): PA modulates synaptic plasticity and energy metabolism via mTOR and PKC pathways, PG is vital for mitochondrial membrane integrity and oxidative phosphorylation, and LPE accumulation signifies enhanced membrane degradation and neuroinflammation (Foster, 2013; Liu et al., 2023). Under HFD conditions, this metabolic axis shifts toward sphingolipid dysregulation, specifically involving the unique increase in Cer-BS d27:2. These findings delineate a coherent ‘microbiota–lipid metabolism–brain’ association, suggesting that HFD acts as a metabolic amplifier that creates a ‘latent vulnerability’ to stress. Rather than a direct behavioral driver in the short term, this HFD-driven reprogramming suggests a potential pathological link from dietary input to neurological deficit, even before such changes fully manifest as statistically divergent behavioral despair.

## 5 Conclusion

In summary, this study constructed a robust multi-omics framework to elucidate the crucial role of lipid signaling in the pathogenesis of CUMS-induced depression and HFD-exacerbated depression. Our findings revealed a hierarchical mechanism within the gut microbiota-lipid-brain axis:

The basal neurobehavioral deficit and pathologic impairment induced by CUMS are primarily associated with the disruption of glycerophospholipid metabolism. This disruption is specifically mediated by metabolites like PA, PG, and LPE, which may contribute to gut microbial dysbiosis, including the depletion of *Bacteroides thetaiotaomicron* and proliferation of *Terrisporobacter glycolicus*, and trigger anxiety-like and depression-like behaviors.

**Figure 8:**
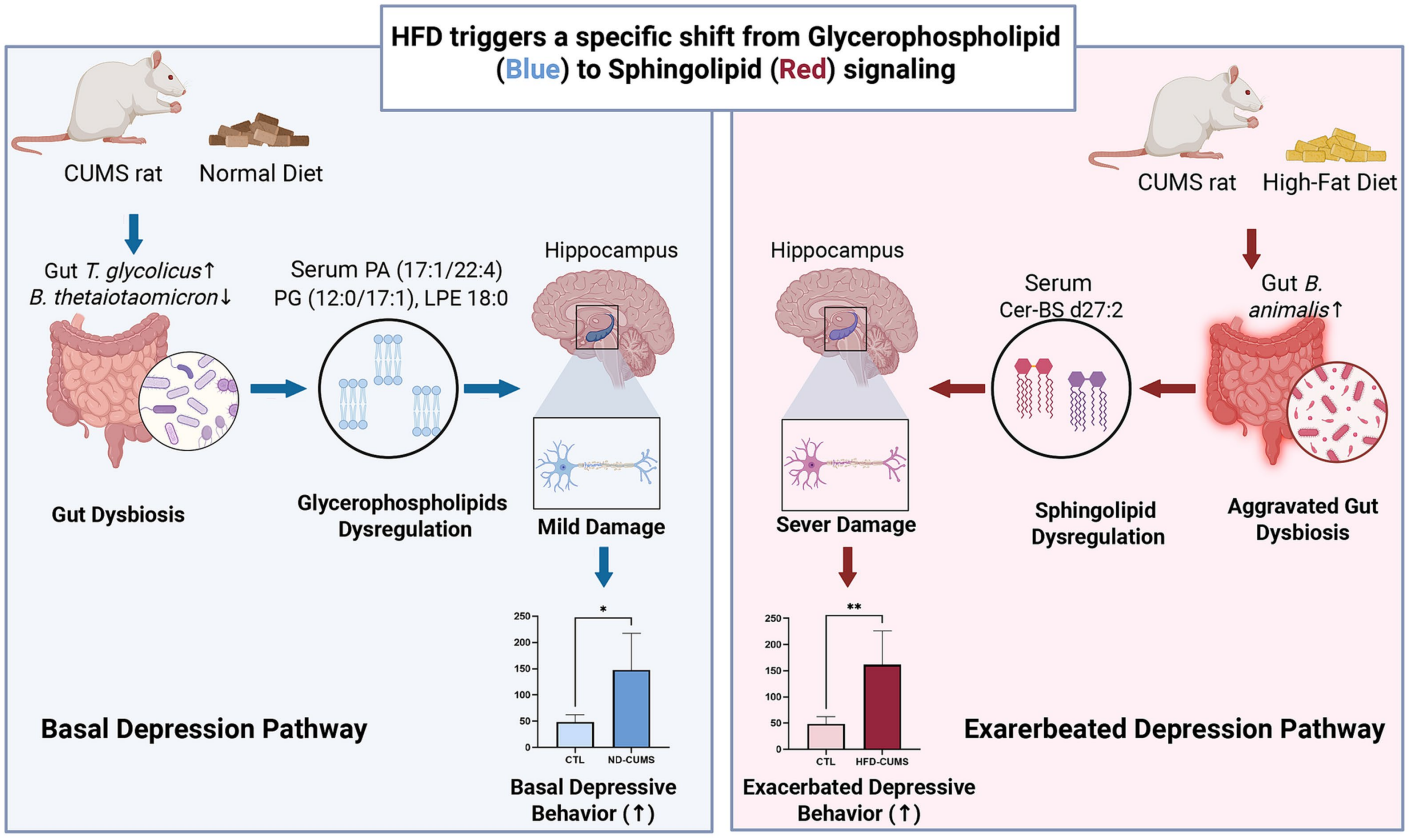
HFD shifts the driver of depressive behavior from glycerophospholipids to sphingolipids via qualitative microbial reprogramming. The schematic illustrates the distinct hierarchical lipid signaling pathways underlying basal versus exacerbated depression. Left panel (Blue): Under a normal diet (ND), CUMS induces basal gut dysbiosis characterized by a decrease in *Bacteroides thetaiotaomicron* and an increase in *Terrisporobacter glycolicus*. This microbial imbalance is associated with the dysregulation of glycerophospholipid metabolism, specifically affecting PA 17:1/22:4, PG 12:0/17:1, and LPE 18:0. This pathway is linked to mild hippocampal neuronal damage and basal depressive-like behaviors. Right panel (Red): HFD intervention exacerbates the pathology by shifting the gut microbiota profile such as the enrichment of *Bifidobacterium animalis*. This HFD-driven shift is characterized by a transition to sphingolipid metabolism disruption, marked by the dysregulation of Ceramide-BS d27:2. Consequently, this pathway leads to severe neuronal damage and a trend toward exacerbated depressive-like phenotypes. Created in BioRender. Cao, Y. (2026) https://BioRender.com/pd4jwm3.

The HFD exacerbates this vulnerability by uniquely amplifying the disruption of the sphingolipid metabolism. This HFD-specific aggravation is precisely marked by the sphingolipid Cer-BS d27:2, which strongly supports a lipid-mediated gut-brain pathway linking microbial reprogramming, such as the *Bifidobacterium* genus, to exacerbated stress susceptibility (Figure 8).

Despite its many strengths, this study is subject to limitations inherent to its correlational design in a rodent model. Rigorous causal validation through interventional approaches such as microbiota transplantation or metabolite manipulation, such as fecal microbiota transplantation or targeted lipid metabolite manipulation, is essential. Future translational research should integrate longitudinal multi-omics with in-human cohorts to map the precise temporal dynamics of two distinct lipid signaling networks and validate metabolic remodeling strategies as feasible therapeutic interventions.

## Data Availability

The data presented in the study are deposited in the China National Center for Bioinformation (CNCB) repository, under BioProject number PRJCA060442 and GSA accession number CRA040336.
