# Supplementary material for: High-fat diet-driven gut microbial sphingolipid metabolic reprogramming is associated with stress susceptibility in CUMS rats
Source: Front Microbiol. 2026 Apr 1;17:1802003. doi: 10.3389/fmicb.2026.1802003 (PMC13082169; doi:10.3389/fmicb.2026.1802003)
Supplement: Supplementary file 1 [file Data_Sheet_1.docx]

**High-Fat Diet-Driven Gut Microbial Sphingolipid Metabolic Reprogramming Is Associated with Stress Susceptibility in CUMS Rats**

**Supplementary table 1. The microbiota associated with CUMS-induced depression**

| Speices | ND-CUMS vs. CTL | | HFD CUMS vs. CTL | |
| --- | --- | --- | --- | --- |
|  | Fold change | p | Fold change | p |
| *Acetivibrio ethanolgignens* | 2.61 | 0.016 | 4.11 | 0.008 |
| *Actinobaculum sp. oral taxon 183* | 2.86 | 0.016 | 2.67 | 0.032 |
| *Anaerostipes sp. 3 2 56FAA* | 2.37 | 0.008 | 2.59 | 0.008 |
| *Bacteroides dorei* | 0.39 | 0.008 | 0.46 | 0.016 |
| *Bacteroides eggerthii CAG:109* | 0.47 | 0.016 | 0.27 | 0.008 |
| *Bacteroides sartorii* | 0.46 | 0.008 | 0.31 | 0.008 |
| *Bacteroides sp. 3 1 13* | 0.50 | 0.008 | 0.32 | 0.008 |
| *Bacteroides sp. 3 1 33FAA* | 0.36 | 0.008 | 0.38 | 0.008 |
| *Bacteroides sp. 3 1 40A* | 0.25 | 0.008 | 0.27 | 0.008 |
| *Bacteroides stercoris CAG:120* | 0.36 | 0.008 | 0.33 | 0.008 |
| *Bacteroides thetaiotaomicron* | 0.27 | 0.008 | 0.21 | 0.008 |
| *Butyrivibrio crossotus* | 3.17 | 0.008 | 4.70 | 0.008 |
| *Butyrivibrio crossotus CAG:259* | 3.77 | 0.008 | 5.94 | 0.008 |
| *Butyrivibrio sp. AD3002* | 4.45 | 0.008 | 4.44 | 0.008 |
| *Butyrivibrio sp. CAG:318* | 6.09 | 0.032 | 14.48 | 0.032 |
| *Butyrivibrio sp. FCS014* | 3.81 | 0.008 | 3.59 | 0.008 |
| *Butyrivibrio sp. LB2008* | 5.98 | 0.008 | 5.70 | 0.008 |
| *Butyrivibrio sp. MB2005* | 3.65 | 0.008 | 3.71 | 0.008 |
| *Butyrivibrio sp. NC3005* | 2.70 | 0.008 | 4.72 | 0.008 |
| *Butyrivibrio sp. WCD2001* | 6.24 | 0.008 | 7.08 | 0.016 |
| *Caloramator sp. ALD01* | 0.12 | 0.008 | 0.14 | 0.008 |
| *Candidatus Methanomethylophilus alvus* | 32.66 | 0.045 | 102.76 | 0.045 |
| *Carnobacterium divergens* | 0.18 | 0.008 | 0.09 | 0.011 |
| *Catonella morbi* | 2.64 | 0.008 | 3.13 | 0.008 |
| *Clostridium glycyrrhizinilyticum* | 2.52 | 0.032 | 2.96 | 0.032 |
| *Clostridium sp. CAG:127* | 4.22 | 0.008 | 6.82 | 0.008 |
| *Clostridium sp. CAG:264* | 3.15 | 0.008 | 4.09 | 0.008 |
| *Clostridium sp. CAG:411* | 3.72 | 0.008 | 4.67 | 0.008 |
| *Clostridium sp. KLE 1755* | 3.28 | 0.008 | 2.95 | 0.008 |
| *Clostridium sp. KNHs209* | 4.24 | 0.008 | 4.02 | 0.008 |
| *Clostridium sp. L2-50* | 3.54 | 0.008 | 5.03 | 0.008 |
| *Clostridium sp. Maddingley MBC34-26* | 3.69 | 0.008 | 3.48 | 0.032 |
| *Clostridium sp. SY8519* | 2.77 | 0.008 | 3.48 | 0.016 |
| *Coprococcus eutactus* | 3.88 | 0.008 | 3.90 | 0.008 |
| *Desulfotomaculum hydrothermale* | 3.38 | 0.008 | 4.43 | 0.008 |
| *Eisenbergiella tayi* | 3.50 | 0.008 | 3.83 | 0.008 |
| *Eubacterium eligens* | 3.24 | 0.032 | 3.97 | 0.008 |
| *Eubacterium eligens CAG:72* | 4.99 | 0.008 | 4.02 | 0.008 |
| *Eubacterium hallii* | 2.21 | 0.016 | 2.84 | 0.008 |
| *Eubacterium plexicaudatum* | 3.36 | 0.016 | 4.72 | 0.008 |
| *Eubacterium ramulus* | 2.28 | 0.016 | 3.34 | 0.008 |
| *Eubacterium rectale* | 2.68 | 0.008 | 5.10 | 0.008 |
| *Eubacterium sp. CAG:156* | 2.09 | 0.016 | 2.89 | 0.032 |
| *Eubacterium sp. CAG:161* | 2.08 | 0.016 | 2.97 | 0.016 |
| *Eubacterium sp. CAG:248* | 4.38 | 0.008 | 6.91 | 0.008 |
| *Eubacterium sp. CAG:252* | 2.33 | 0.032 | 3.88 | 0.008 |
| *Lachnoanaerobaculum saburreum* | 3.18 | 0.016 | 2.90 | 0.008 |
| *Leptospirillum sp. Group III* | 0.39 | 0.008 | 0.28 | 0.034 |
| *Leptotrichia hofstadii* | 2.62 | 0.032 | 2.71 | 0.032 |
| *Mycoplasma californicum* | 44.69 | 0.011 | 69.15 | 0.011 |
| *Odoribacter sp. CAG:788* | 0.45 | 0.008 | 0.39 | 0.008 |
| *Oribacterium asaccharolyticum* | 2.87 | 0.032 | 2.82 | 0.032 |
| *Oribacterium sp. FC2011* | 2.23 | 0.032 | 2.42 | 0.032 |
| *Parabacteroides sp. 20 3* | 0.40 | 0.008 | 0.38 | 0.008 |
| *Parabacteroides sp. D13* | 0.43 | 0.008 | 0.41 | 0.008 |
| *Pedobacter glucosidilyticus* | 0.08 | 0.016 | 0.07 | 0.008 |
| *Prevotella dentalis* | 0.48 | 0.008 | 0.40 | 0.008 |
| *Prevotella stercorea CAG:629* | 0.38 | 0.008 | 0.40 | 0.016 |
| *Pseudobutyrivibrio ruminis* | 4.01 | 0.008 | 4.83 | 0.008 |
| *Pseudobutyrivibrio sp. MD2005* | 6.56 | 0.008 | 6.28 | 0.008 |
| *Rhodonellum psychrophilum* | 114.97 | 0.012 | 98.51 | 0.012 |
| *Roseburia faecis* | 4.27 | 0.008 | 8.18 | 0.008 |
| *Roseburia hominis* | 6.65 | 0.008 | 12.24 | 0.008 |
| *Roseburia intestinalis* | 5.58 | 0.008 | 16.19 | 0.008 |
| *Roseburia intestinalis CAG:13* | 2.46 | 0.008 | 7.91 | 0.008 |
| *Roseburia inulinivorans* | 4.11 | 0.008 | 8.52 | 0.008 |
| *Roseburia inulinivorans CAG:15* | 2.53 | 0.008 | 3.48 | 0.016 |
| *Roseburia sp. CAG:100* | 4.56 | 0.008 | 4.69 | 0.008 |
| *Roseburia sp. CAG:182* | 3.09 | 0.008 | 5.03 | 0.008 |
| *Roseburia sp. CAG:197* | 4.79 | 0.016 | 8.36 | 0.008 |
| *Roseburia sp. CAG:380* | 4.18 | 0.016 | 5.73 | 0.008 |
| *Ruminococcus lactaris* | 2.12 | 0.016 | 2.33 | 0.008 |
| *Ruminococcus sp. CAG:9* | 3.75 | 0.008 | 5.54 | 0.008 |
| *Serratia fonticola* | 0.17 | 0.008 | 0.06 | 0.008 |
| *Sphingobacterium spiritivorum* | 0.39 | 0.032 | 0.14 | 0.012 |
| *Streptococcus massiliensis* | 5.94 | 0.032 | 10.81 | 0.008 |
| *Terrisporobacter glycolicus* | 2.37 | 0.016 | 2.91 | 0.008 |
| *Treponema azotonutricium* | 6.23 | 0.032 | 5.64 | 0.008 |
| *Treponema bryantii* | 3.24 | 0.032 | 4.46 | 0.008 |
| *Treponema sp. JC4* | 3.36 | 0.008 | 2.89 | 0.032 |
| *uncultured Prevotella sp.* | 0.22 | 0.020 | 0.17 | 0.020 |
| *Allobaculum stercoricanis* | 23.56 | 0.008 | 12.99 | 0.032 |
| *Bacteroides dorei CAG:222* | 0.20 | 0.008 | 0.37 | 0.016 |
| *Bacteroides massiliensis* | 0.32 | 0.008 | 0.47 | 0.032 |
| *Bacteroides vulgatus CAG:6* | 0.09 | 0.008 | 0.30 | 0.032 |
| *Butyrivibrio sp. AE2032* | 2.98 | 0.008 | 2.36 | 0.032 |
| *Butyrivibrio sp. AE3003* | 7.35 | 0.016 | 5.37 | 0.032 |
| *Butyrivibrio sp. VCB2001* | 8.84 | 0.008 | 5.22 | 0.008 |
| *Chlorobium ferrooxidans* | 34.67 | 0.008 | 22.14 | 0.008 |
| *Clostridium sp. ND2* | 0.24 | 0.008 | 0.44 | 0.032 |
| *Enterococcus raffinosus* | 24.83 | 0.034 | 13.86 | 0.011 |
| *Flavobacterium daejeonense* | 0.25 | 0.008 | 0.38 | 0.016 |
| *Lachnoclostridium phytofermentans* | 4.88 | 0.008 | 3.22 | 0.008 |
| *Lactobacillus gastricus* | 44.77 | 0.012 | 27.39 | 0.012 |
| *Lactococcus garvieae* | 3.24 | 0.032 | 2.34 | 0.016 |
| *Nocardia otitidiscaviarum* | 23.23 | 0.012 | 14.02 | 0.021 |
| *Paenibacillus harenae* | 9.59 | 0.008 | 5.07 | 0.016 |
| *Paenibacillus sp. Y24* | 88.44 | 0.011 | 52.10 | 0.011 |
| *Parabacteroides sp. D25* | 0.13 | 0.008 | 0.26 | 0.016 |
| *Roseburia sp. CAG:303* | 8.11 | 0.008 | 4.56 | 0.008 |
| *Roseburia sp. CAG:45* | 6.69 | 0.008 | 4.41 | 0.016 |
| *Sphingobacterium sp. ACCC 05744* | 0.28 | 0.008 | 0.36 | 0.032 |
| *Streptococcus infantarius* | 0.24 | 0.008 | 0.36 | 0.008 |
| *Streptococcus pasteurianus* | 39.11 | 0.008 | 29.76 | 0.008 |
| *Syntrophomonas wolfei* | 3.10 | 0.016 | 2.33 | 0.032 |
| *Virgibacillus pantothenticus* | 0.15 | 0.012 | 0.29 | 0.036 |

p: Wilcoxon rank-sum test.

**Supplementary table 2. The microbiota associated with HFD-exacerbated depression**

| Speices | HFD CUMS vs. CTL | | HFD CUMS vs. ND-CUMS | |
| --- | --- | --- | --- | --- |
|  | Fold change | p | Fold change | p |
| *Actinobacillus minor* | 0.08 | 0.012 | 0.10 | 0.012 |
| *Alicyclobacillus macrosporangiidus* | 16.66 | 0.016 | 14.94 | 0.021 |
| *Alteromonas australica* | 49.04 | 0.020 | 10.33 | 0.018 |
| *Anaerobiospirillum succiniciproducens* | 6.91 | 0.008 | 3.75 | 0.008 |
| *Bacillus alcalophilus* | 8.69 | 0.032 | 8.22 | 0.016 |
| *Bacillus altitudinis* | 23.48 | 0.011 | 11.81 | 0.012 |
| *Bacillus pumilus* | 3.39 | 0.008 | 3.22 | 0.008 |
| *Bifidobacterium animalis* | 8.72 | 0.008 | 4.11 | 0.008 |
| *Bifidobacterium longum* | 6.60 | 0.008 | 4.15 | 0.008 |
| *Bifidobacterium magnum* | 29.30 | 0.008 | 13.49 | 0.008 |
| *Bifidobacterium sp. AGR2158* | 161.12 | 0.012 | 169.39 | 0.008 |
| *Candidatus Amoebophilus asiaticus* | 4.97 | 0.008 | 3.98 | 0.032 |
| *Chlorobium limicola* | 0.01 | 0.010 | 0.01 | 0.010 |
| *Clostridium novyi* | 4.87 | 0.008 | 2.25 | 0.008 |
| *Clostridium sp. DMHC 10* | 3.50 | 0.032 | 2.09 | 0.032 |
| *Coprobacillus sp. CAG:235* | 3.81 | 0.008 | 2.98 | 0.008 |
| *Deinococcus pimensis* | 7.05 | 0.008 | 5.86 | 0.016 |
| *Desulfitobacterium sp. PCE1* | 6.13 | 0.016 | 5.86 | 0.008 |
| *Desulfotomaculum acetoxidans* | 2.64 | 0.016 | 2.40 | 0.032 |
| *Dorea formicigenerans CAG:28* | 4.52 | 0.008 | 2.44 | 0.008 |
| *Enterococcus pallens* | 3.24 | 0.008 | 2.11 | 0.032 |
| *Enterorhabdus caecimuris* | 4.55 | 0.008 | 3.71 | 0.008 |
| *Eubacterium sp. AB3007* | 0.40 | 0.032 | 0.40 | 0.032 |
| *Eubacterium sp. CAG:146* | 2.86 | 0.008 | 2.16 | 0.032 |
| *Fibrella aestuarina* | 0.02 | 0.045 | 0.02 | 0.045 |
| *Flavobacterium aquatile* | 0.07 | 0.008 | 0.14 | 0.008 |
| *Lactobacillus mucosae* | 0.11 | 0.016 | 0.17 | 0.008 |
| *Luteimonas abyssi* | 0.03 | 0.010 | 0.06 | 0.045 |
| *Lutibaculum baratangense* | 58.24 | 0.012 | 32.49 | 0.012 |
| *Lysinibacillus fusiformis* | 20.30 | 0.008 | 4.36 | 0.032 |
| *Marinobacter salarius* | 29.41 | 0.045 | 7.89 | 0.045 |
| *Megasphaera sp. UPII 135-E* | 9.78 | 0.032 | 9.08 | 0.008 |
| *Methanococcus aeolicus* | 5.15 | 0.008 | 3.96 | 0.008 |
| *Methylococcus capsulatus* | 41.52 | 0.012 | 12.36 | 0.011 |
| *Moraxella caprae* | 0.17 | 0.012 | 0.29 | 0.021 |
| *Paenibacillus sonchi* | 6.65 | 0.016 | 5.89 | 0.016 |
| *Paenibacillus sp. PAMC 26794* | 34.78 | 0.008 | 2.94 | 0.008 |
| *Pediococcus claussenii* | 20.24 | 0.016 | 10.33 | 0.036 |
| *Photorhabdus temperata* | 6.43 | 0.016 | 5.22 | 0.032 |
| *Porphyromonas gingivicanis* | 0.40 | 0.016 | 0.42 | 0.032 |
| *Rhodocista sp. AAP38* | 0.02 | 0.045 | 0.04 | 0.031 |
| *Roseburia intestinalis* | 16.19 | 0.008 | 2.90 | 0.032 |
| *Roseburia intestinalis CAG:13* | 7.91 | 0.008 | 3.21 | 0.008 |
| *Roseburia inulinivorans* | 8.52 | 0.008 | 2.07 | 0.016 |
| *Ruminococcus sp. JC304* | 2.73 | 0.032 | 2.54 | 0.032 |
| *Serratia fonticola* | 0.06 | 0.008 | 0.36 | 0.008 |
| *Shigella sonnei* | 0.05 | 0.016 | 0.17 | 0.032 |
| *Sphingobacterium thalpophilum* | 0.12 | 0.012 | 0.19 | 0.012 |
| *Staphylococcus pseudintermedius* | 8.41 | 0.034 | 10.55 | 0.032 |
| *Staphylococcus saprophyticus* | 0.03 | 0.045 | 0.04 | 0.045 |
| *Streptomyces sp. NRRL WC-3719* | 0.04 | 0.045 | 0.04 | 0.045 |
| *Thermicanus aegyptius* | 19.77 | 0.008 | 7.56 | 0.032 |
| *Turicibacter sanguinis* | 4.36 | 0.008 | 3.28 | 0.032 |
| *Turicibacter sp. HGF1* | 7.29 | 0.008 | 4.57 | 0.032 |
| *Zunongwangia profunda* | 0.27 | 0.032 | 0.46 | 0.032 |
| *Aerococcus christensenii* | 3.16 | 0.016 | 8.73 | 0.008 |
| *Alcaligenes faecalis* | 0.04 | 0.045 | 0.02 | 0.010 |
| *Alistipes timonensis* | 0.33 | 0.008 | 0.31 | 0.016 |
| *Anaerosalibacter sp. ND1* | 0.36 | 0.032 | 0.27 | 0.008 |
| *Bacillus sp. J37* | 5.77 | 0.016 | 6.45 | 0.016 |
| *Bifidobacterium choerinum* | 558.68 | 0.032 | 839.05 | 0.036 |
| *Clostridium sp. NCR* | 3.70 | 0.008 | 4.40 | 0.016 |
| *Corynebacterium sp. JCB* | 0.07 | 0.034 | 0.04 | 0.020 |
| *Desulfuromonas sp. TF* | 0.05 | 0.018 | 0.02 | 0.010 |
| *Eggerthella sp. 1 3 56FAA* | 2.53 | 0.032 | 3.65 | 0.016 |
| *Enterococcus asini* | 3.07 | 0.008 | 4.15 | 0.008 |
| *Formosa agariphila* | 0.08 | 0.012 | 0.05 | 0.036 |
| *Solirubrobacter sp. URHD0082* | 8.46 | 0.008 | 12.50 | 0.011 |

p: Wilcoxon rank-sum test.

**Supplementary table 3. The metabolites associated with CUMS-induced depression**

| ID |  | ND-CUMS vs. CTL | | | HFD-CUMS vs. CTL | | |
| --- | --- | --- | --- | --- | --- | --- | --- |
|  | Metabolites | Fold change | p | VIP | Fold change | p | VIP |
| N4359 | Spirotaccagenin | 0.32 | 0.031 | 1.56 | 0.28 | 0.032 | 1.36 |
| N2751 | 5(S)-Hpete | 4.25 | 0.004 | 2.01 | 5.61 | 0.027 | 1.20 |
| P4947 | Sufentanil | 0.12 | 0.000 | 2.22 | 0.04 | 0.000 | 2.27 |
| P6094 | Cinegalline | 0.16 | 0.000 | 2.37 | 0.06 | 0.000 | 2.40 |
| P6830 | NCGC00160314-01 | 0.20 | 0.014 | 1.92 | 0.03 | 0.001 | 2.15 |
| P4473 | Piperundecalidine | 0.22 | 0.007 | 2.02 | 0.07 | 0.000 | 2.22 |
| P6967 | MLS001142777-01 | 0.24 | 0.025 | 1.87 | 0.05 | 0.001 | 2.15 |
| P5710 | Spironolactone | 0.27 | 0.005 | 1.96 | 0.10 | 0.001 | 2.15 |
| P7387 | MLS001142425-01 | 0.27 | 0.002 | 2.15 | 0.14 | 0.000 | 2.27 |
| P3492 | Pencycuron | 0.29 | 0.008 | 1.95 | 0.17 | 0.001 | 2.12 |
| P10676 | Daphnoline | 0.40 | 0.024 | 2.00 | 0.36 | 0.008 | 1.92 |
| P19442 | Thalassospiramide B | 0.44 | 0.031 | 1.82 | 0.33 | 0.004 | 1.94 |
| P8633 | Taurohyocholate | 0.46 | 0.035 | 1.92 | 0.41 | 0.012 | 1.79 |
| P5076 | Sparfloxacin | 0.47 | 0.001 | 1.99 | 0.49 | 0.008 | 1.86 |
| P13149 | PA(12:0/21:0) | 0.47 | 0.013 | 2.09 | 0.47 | 0.013 | 1.66 |
| P5544 | Risperidone | 0.48 | 0.044 | 1.73 | 0.41 | 0.010 | 1.74 |
| P7773 | lysoPE 18:0 | 2.01 | 0.016 | 1.70 | 2.29 | 0.005 | 2.21 |
| P12703 | DG(18:3/20:0/0:0) | 2.04 | 0.017 | 2.10 | 2.16 | 0.001 | 2.09 |
| P4873 | Acetylleucyl-leucyl-norleucinal | 2.08 | 0.012 | 2.07 | 2.37 | 0.000 | 1.91 |
| P18667 | SQDG 22:4/22:6 | 2.10 | 0.021 | 2.03 | 2.18 | 0.000 | 2.17 |
| P18242 | TAG 18:5/18:5/20:5 | 2.12 | 0.037 | 1.92 | 2.28 | 0.019 | 1.63 |
| P17699 | DGDG 30:5 | 2.19 | 0.049 | 1.79 | 2.39 | 0.009 | 1.71 |
| P7123 | Glycocholate | 2.19 | 0.003 | 2.16 | 2.73 | 0.024 | 2.18 |
| P3454 | 10-HDoHE | 2.25 | 0.032 | 1.90 | 2.84 | 0.043 | 2.06 |
| P1415 | 3,4,5-Trimethoxycinnamic acid | 2.48 | 0.015 | 2.10 | 3.12 | 0.000 | 2.14 |
| P3106 | Botrydial | 2.71 | 0.049 | 1.88 | 2.93 | 0.000 | 2.20 |
| P17150 | plasmenyl-PC 40:6 | 2.72 | 0.001 | 2.32 | 2.78 | 0.000 | 2.09 |
| P15658 | HexCer_BS d16:3/23:5 | 2.80 | 0.007 | 2.15 | 3.36 | 0.001 | 1.97 |
| P15026 | SM 35:2 | 3.05 | 0.003 | 2.15 | 5.08 | 0.000 | 2.17 |
| P3004 | Mesterolone | 3.35 | 0.013 | 2.02 | 5.49 | 0.047 | 1.96 |
| P16733 | Licoricesaponin E2 | 3.61 | 0.001 | 2.23 | 3.28 | 0.011 | 1.89 |
| P19035 | GlcADG 26:1/24:4 | 4.05 | 0.009 | 2.13 | 3.83 | 0.000 | 2.09 |
| P4233 | Prednisone | 4.23 | 0.005 | 2.21 | 4.81 | 0.021 | 2.14 |
| P13596 | SM 31:3 | 4.46 | 0.040 | 1.79 | 4.90 | 0.000 | 2.46 |
| P4694 | Picotamide | 4.52 | 0.006 | 2.15 | 5.04 | 0.010 | 2.30 |
| P1399 | α-Vetivone | 4.77 | 0.006 | 2.22 | 6.22 | 0.000 | 2.26 |
| P5080 | Kalihinol A | 4.78 | 0.011 | 2.15 | 6.12 | 0.042 | 1.84 |
| P16136 | SQDG 16:0_16:0 | 5.25 | 0.016 | 2.09 | 7.31 | 0.000 | 2.13 |
| P8278 | Eudesobovatol A | 5.37 | 0.007 | 2.23 | 6.56 | 0.001 | 2.00 |
| P6585 | Westcort | 5.40 | 0.005 | 2.26 | 5.88 | 0.000 | 2.49 |
| P2583 | Retinal | 5.45 | 0.005 | 2.19 | 8.14 | 0.034 | 2.02 |
| P3861 | Q27149131 | 5.54 | 0.006 | 2.19 | 7.97 | 0.046 | 1.93 |
| P11907 | Rakicidin B | 5.54 | 0.005 | 2.22 | 7.41 | 0.000 | 2.01 |
| P19034 | PS 50:3 | 5.78 | 0.016 | 2.10 | 8.17 | 0.000 | 2.20 |
| P2157 | Vetiveryl acetate | 5.99 | 0.010 | 2.03 | 6.73 | 0.009 | 2.11 |
| P9007 | PAF C-16-d4 | 6.04 | 0.021 | 1.90 | 6.76 | 0.000 | 2.00 |
| P2950 | Eicosapentaenoic acid | 6.10 | 0.005 | 2.22 | 9.13 | 0.033 | 2.02 |
| P3433 | Oleoylethanolamide | 7.02 | 0.018 | 1.94 | 8.07 | 0.001 | 2.37 |
| P19344 | AcylGlcADG 18:4/20:0/14:1 | 7.30 | 0.011 | 2.15 | 9.67 | 0.000 | 2.41 |
| P19995 | DGDG 27:0/22:5 | 7.77 | 0.010 | 2.19 | 9.97 | 0.000 | 2.06 |
| P13592 | PG(12:0/17:1) | 8.72 | 0.005 | 2.26 | 10.07 | 0.000 | 2.09 |
| P19009 | GlcADG 26:2/24:4 | 9.26 | 0.019 | 2.06 | 9.50 | 0.000 | 2.46 |
| P17375 | MGDG 40:6 | 10.90 | 0.017 | 2.08 | 19.51 | 0.000 | 2.07 |
| P10105 | Baliospermin | 15.83 | 0.006 | 2.27 | 18.36 | 0.000 | 2.07 |
| P19161 | HBMP 16:4/18:0/18:2 | 17.51 | 0.016 | 2.11 | 19.36 | 0.000 | 2.08 |
| P18753 | DGDG 36:1 | 22.03 | 0.008 | 2.23 | 26.31 | 0.000 | 2.13 |
| P14921 | LMGP02040016 | 23.31 | 0.027 | 2.02 | 39.47 | 0.000 | 2.35 |
| P18555 | TAG 16:5/21:4/22:7 | 23.46 | 0.007 | 2.21 | 30.43 | 0.000 | 2.28 |
| P18718 | PS 47:2 | 27.56 | 0.009 | 2.21 | 33.36 | 0.000 | 2.18 |
| P15020 | PA(17:1/22:4) | 37.32 | 0.009 | 2.19 | 55.13 | 0.000 | 2.25 |
| P17303 | PC 39:3 | 59.04 | 0.012 | 2.14 | 68.98 | 0.000 | 2.3042 |
| P18196 | TAG 16:3/18:5/22:7 | 122.98 | 0.007 | 2.22 | 147.42 | 0.000 | 2.41292 |

p: Student’s t-test.

**Supplementary table 4. The metabolites associated with HFD-exacerbated depression**

| ID |  | HFD-CUMS vs. CTL | | | HFD-CUMS vs. ND-CUMS | | |
| --- | --- | --- | --- | --- | --- | --- | --- |
|  | Metabolites | FC | P | VIP | FC | P | VIP |
| N5936 | N4-Acetylcytidine | 0.30 | 0.018 | 2.17 | 2.32 | 0.033 | 1.91 |
| N5714 | Prupaside | 0.49 | 0.039 | 1.05 | 1.91 | 0.029 | 1.36 |
| N4047 | 3,7,12,19-THCA | 7.07 | 0.013 | 1.49 | 0.24 | 0.027 | 1.46 |
| N3385 | Persenone A | 7.41 | 0.004 | 1.47 | 0.23 | 0.008 | 1.46 |
| N4048 | 3,4,7,12-THCA | 8.34 | 0.024 | 1.47 | 0.23 | 0.043 | 1.47 |
| N3384 | [12]-Gingerol | 10.36 | 0.011 | 1.53 | 0.23 | 0.026 | 1.48 |
| P8123 | Taurochenodeoxycholate | 0.44 | 0.021 | 1.49 | 0.57 | 0.030 | 1.45 |
| P8122 | Taurodeoxycholate | 0.44 | 0.015 | 1.76 | 0.20 | 0.029 | 2.06 |
| P15110 | PA(17:2/22:1) | 0.49 | 0.012 | 1.55 | 0.44 | 0.008 | 2.00 |
| P18361 | Hoiamide A | 0.58 | 0.028 | 1.43 | 0.64 | 0.012 | 1.27 |
| P15824 | PC 34:2 | 0.59 | 0.031 | 1.39 | 0.46 | 0.016 | 1.96 |
| P1988 | Dyphylline | 0.62 | 0.027 | 1.39 | 0.64 | 0.029 | 1.46 |
| P15744 | plasmenyl-PE 38:3 | 0.65 | 0.014 | 1.26 | 0.65 | 0.010 | 1.32 |
| P15590 | Arachidonoyl PAF C-16 | 0.66 | 0.032 | 1.02 | 0.65 | 0.032 | 1.18 |
| P2470 | 9(S)-HODE | 1.54 | 0.029 | 1.68 | 1.66 | 0.027 | 2.13 |
| P5937 | Elaidic carnitine | 1.80 | 0.012 | 1.66 | 1.67 | 0.009 | 1.94 |
| P381 | 4-Chlorobenzaldehyde | 2.01 | 0.041 | 1.65 | 2.34 | 0.012 | 2.37 |
| P2451 | L-368899 | 2.09 | 0.036 | 1.74 | 1.94 | 0.036 | 2.05 |
| P12565 | PA(14:0/18:3) | 2.09 | 0.026 | 1.61 | 1.61 | 0.045 | 1.81 |
| P13792 | Cer-NS d21:2/24:2 | 2.10 | 0.030 | 1.73 | 1.84 | 0.030 | 2.02 |
| P6785 | Cer-BS d14:2/13:0 | 2.12 | 0.014 | 1.80 | 1.96 | 0.008 | 2.08 |
| P1915 | Diaporthin | 3.48 | 0.034 | 1.82 | 3.36 | 0.037 | 2.02 |
| P9453 | LMST05040008 | 4.12 | 0.000 | 2.03 | 3.11 | 0.000 | 2.33 |
| P15026 | SM 35:2 | 5.08 | 0.000 | 2.17 | 1.67 | 0.005 | 1.88 |

p: Student’s t-test.


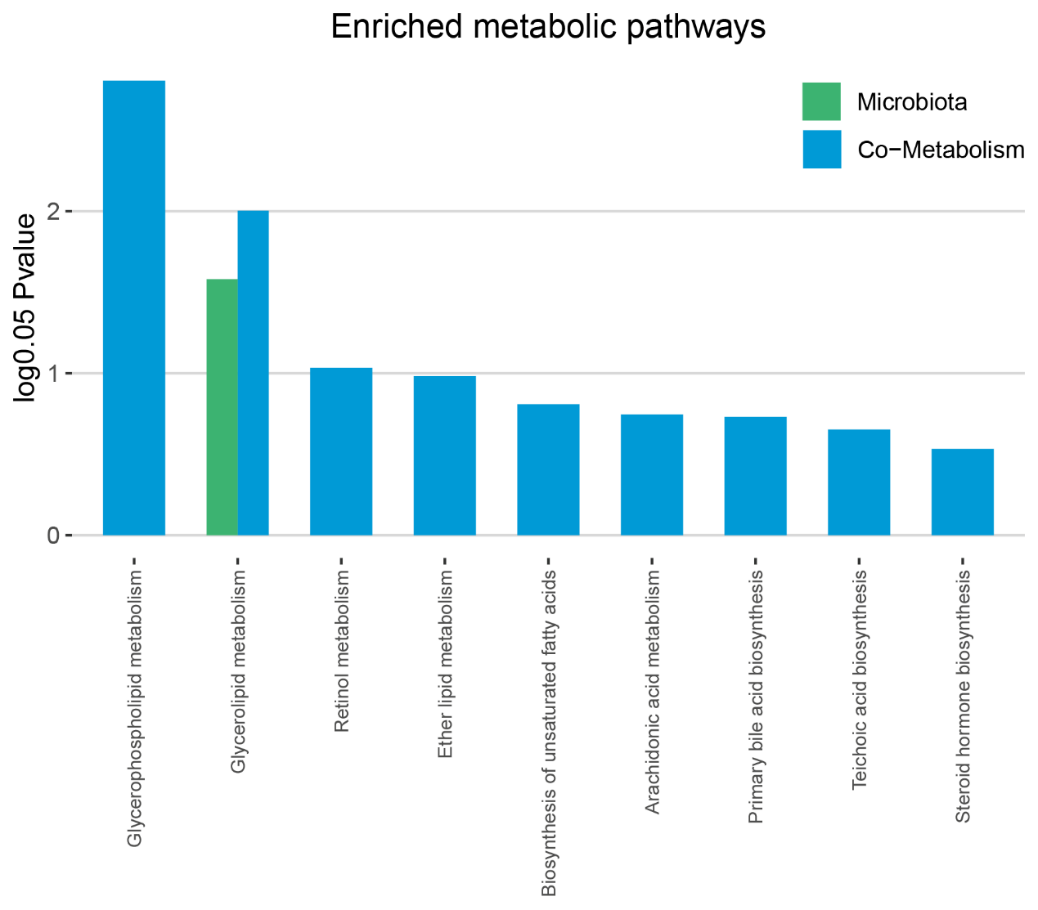


**Supplementary Figure 1. Origin analysis and metabolic pathway enrichment analysis (MPEA) of [(ND‑CUMS vs. CTL) ∩ (HFD‑CUMS vs. CTL)].** MPEA analysis based on subgroup of metabolites from the host, microbiota, and co-metabolism.


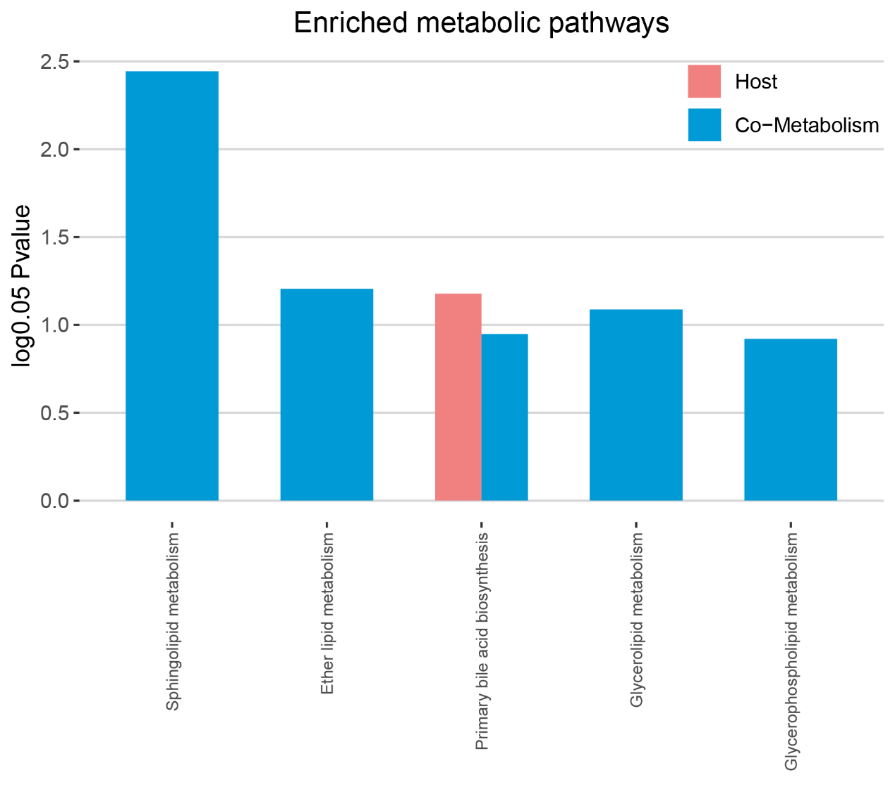


**Supplementary Figure 2. Origin analysis and metabolic pathway enrichment analysis (MPEA) of [(HFD-CUMS vs. ND-CUMS) ∩ (HFD-CUMS vs. CTL)].** MPEA analysis based on subgroup of metabolites from the host, microbiota, and co-metabolism.


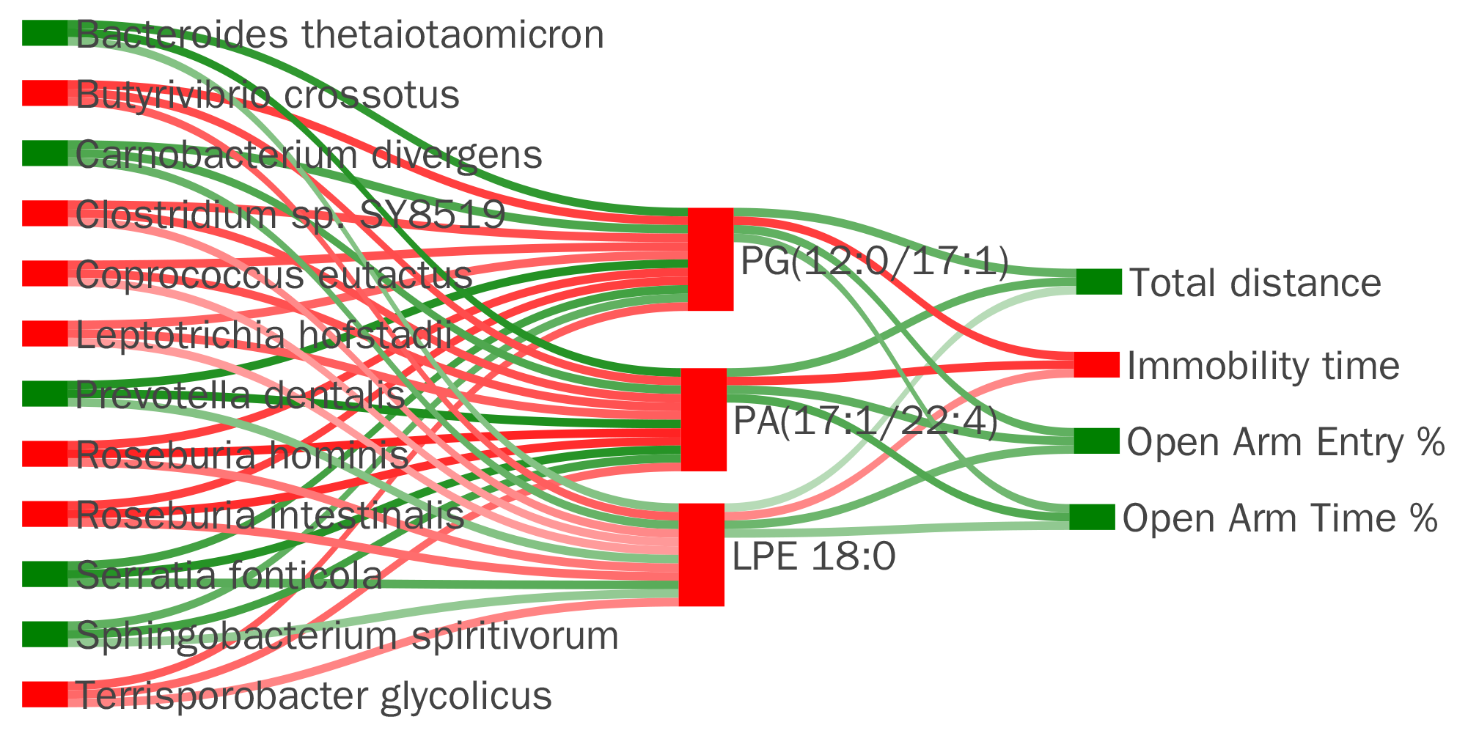


**Supplementary Figure 3.** Sankey diagram illustrating the hierarchical associations between key gut microbial taxa, differential serum metabolites, and behavioral indices in the [(ND‑CUMS vs. CTL) ∩ (HFD‑CUMS vs. CTL)] group. The flow originates from the bacterial genus level (left), transitions through major serum metabolites (middle), and terminates at neurobehavioral readouts (right). The width of the connecting lines represents the magnitude of the correlation coefficient, while the color of the lines indicates the direction of the association (red: positive correlation; green: negative correlation).


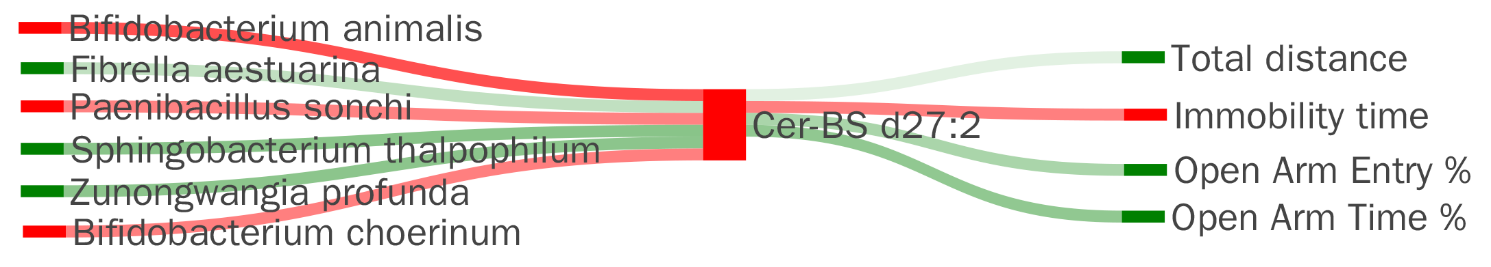


**Supplementary Figure 4.** Sankey diagram illustrating the hierarchical associations between key gut microbial taxa, differential serum metabolites, and behavioral indices in the [(HFD-CUMS vs. ND-CUMS) ∩ (HFD-CUMS vs. CTL)] group. The flow originates from the bacterial genus level (left), transitions through major serum metabolites (middle), and terminates at neurobehavioral readouts (right). The width of the connecting lines represents the magnitude of the correlation coefficient, while the color of the lines indicates the direction of the association (red: positive correlation; green: negative correlation).

**Supplementary table 5**. Mediation effect parameters and stability estimates for the microbiota-metabolite-behavior axis in the [(ND CUMS vs. CTL) ∩ (HFD CUMS vs. CTL)] group.

| Microbiota | Metabolites | Phenotype | ACME | 95% CI  (Lower) | 95% CI  (Upper) | P-value  (Bootstrap) | Stability Rate |
| --- | --- | --- | --- | --- | --- | --- | --- |
| Roseburia intestinalis | PA(17:1/22:4) | OE % | 0.464 | -0.438 | 1.562 | 0.452 | 0.768 |
| Serratia fonticola | PG(12:0/17:1) | OT % | 0.105 | -0.11 | 0.617 | 0.48 | 0.893 |
| Leptotrichia hofstadii | PA(17:1/22:4) | OE % | 0.08 | -0.135 | 0.374 | 0.485 | 0.766 |
| Carnobacterium divergens | LPE 18:0 | Immobility time | 0.086 | -0.185 | 0.441 | 0.496 | 0.786 |
| Carnobacterium divergens | LPE 18:0 | OT % | -0.126 | -0.356 | 0.02 | 0.496 | 0.964 |
| Leptotrichia hofstadii | PG(12:0/17:1) | Immobility time | 0.068 | -0.762 | 0.302 | 0.502 | 0.741 |
| Coprococcus eutactus | LPE 18:0 | Immobility time | -0.068 | -0.703 | 0.144 | 0.51 | 0.759 |
| Coprococcus eutactus | LPE 18:0 | Total distance | 0.051 | -0.183 | 0.749 | 0.514 | 0.731 |
| Serratia fonticola | PA(17:1/22:4) | OT % | 0.063 | -0.204 | 0.445 | 0.519 | 0.759 |
| Bacteroides thetaiotaomicron | PA(17:1/22:4) | OE % | -0.137 | -1.173 | 0.241 | 0.521 | 0.778 |
| Serratia fonticola | PA(17:1/22:4) | OE % | -0.216 | -1.032 | 0.391 | 0.522 | 0.778 |
| Coprococcus eutactus | LPE 18:0 | OE % | 0.055 | -0.174 | 0.531 | 0.523 | 0.721 |
| Clostridium sp. SY8519 | LPE 18:0 | OE % | 0.247 | -0.126 | 1.259 | 0.531 | 0.922 |
| Carnobacterium divergens | LPE 18:0 | Total distance | -0.103 | -0.577 | 0.263 | 0.532 | 0.771 |
| Serratia fonticola | LPE 18:0 | Immobility time | 0.098 | -0.723 | 0.864 | 0.538 | 0.68 |
| Carnobacterium divergens | PG(12:0/17:1) | OT % | 0.034 | -0.069 | 0.272 | 0.542 | 0.819 |
| Coprococcus eutactus | LPE 18:0 | OT % | 0.088 | -0.097 | 0.566 | 0.542 | 0.818 |
| Clostridium sp. SY8519 | LPE 18:0 | Immobility time | -0.192 | -0.781 | 0.347 | 0.543 | 0.854 |
| Butyrivibrio crossotus | PA(17:1/22:4) | OT % | 0.026 | -0.088 | 0.275 | 0.559 | 0.8 |
| Carnobacterium divergens | PA(17:1/22:4) | OE % | -0.047 | -0.328 | 0.276 | 0.562 | 0.563 |
| Butyrivibrio crossotus | PA(17:1/22:4) | OE % | -0.051 | -0.379 | 0.48 | 0.564 | 0.794 |
| Roseburia intestinalis | PG(12:0/17:1) | OT % | -0.148 | -0.948 | 0.465 | 0.567 | 0.726 |
| Clostridium sp. SY8519 | LPE 18:0 | OT % | 0.261 | 0.013 | 0.819 | 0.569 | 0.981 |
| Leptotrichia hofstadii | PG(12:0/17:1) | OT % | -0.032 | -0.248 | 0.359 | 0.574 | 0.737 |
| Coprococcus eutactus | PG(12:0/17:1) | Immobility time | 0.05 | -0.684 | 0.542 | 0.575 | 0.656 |
| Roseburia intestinalis | PG(12:0/17:1) | OE % | 0.346 | -1.16 | 1.457 | 0.577 | 0.634 |
| Coprococcus eutactus | PG(12:0/17:1) | OT % | -0.036 | -0.493 | 0.378 | 0.577 | 0.686 |
| Sphingobacterium spiritivorum | PA(17:1/22:4) | OE % | 0.027 | -0.079 | 0.567 | 0.586 | 0.798 |
| Carnobacterium divergens | PA(17:1/22:4) | OT % | 0.014 | -0.141 | 0.204 | 0.587 | 0.666 |
| Sphingobacterium spiritivorum | PA(17:1/22:4) | OT % | -0.007 | -0.135 | 0.096 | 0.588 | 0.706 |
| Serratia fonticola | LPE 18:0 | OT % | -0.101 | -0.6 | 0.457 | 0.591 | 0.747 |
| Serratia fonticola | PG(12:0/17:1) | Total distance | 0.141 | -0.646 | 0.468 | 0.593 | 0.627 |
| Serratia fonticola | LPE 18:0 | OE % | -0.052 | -0.727 | 0.871 | 0.593 | 0.625 |
| Coprococcus eutactus | PA(17:1/22:4) | OE % | 0.068 | -0.251 | 0.74 | 0.594 | 0.682 |
| Leptotrichia hofstadii | LPE 18:0 | OE % | 0.029 | -0.265 | 0.353 | 0.6 | 0.659 |
| Terrisporobacter glycolicus | PG(12:0/17:1) | OT % | -0.011 | -0.107 | 0.106 | 0.603 | 0.556 |
| Roseburia hominis | PG(12:0/17:1) | OT % | -0.027 | -0.352 | 0.372 | 0.608 | 0.599 |
| Serratia fonticola | PG(12:0/17:1) | Immobility time | -0.101 | -0.392 | 1.042 | 0.61 | 0.619 |
| Leptotrichia hofstadii | LPE 18:0 | Total distance | 0.031 | -0.28 | 0.595 | 0.613 | 0.63 |
| Clostridium sp. SY8519 | LPE 18:0 | Total distance | 0.107 | -0.419 | 0.841 | 0.614 | 0.704 |
| Serratia fonticola | LPE 18:0 | Total distance | -0.044 | -0.608 | 0.765 | 0.614 | 0.596 |
| Leptotrichia hofstadii | LPE 18:0 | Immobility time | -0.041 | -0.531 | 0.321 | 0.619 | 0.664 |
| Leptotrichia hofstadii | LPE 18:0 | OT % | 0.05 | -0.145 | 0.385 | 0.62 | 0.722 |
| Serratia fonticola | PA(17:1/22:4) | Immobility time | -0.095 | -0.438 | 0.774 | 0.627 | 0.632 |
| Serratia fonticola | PA(17:1/22:4) | Total distance | 0.135 | -0.829 | 0.448 | 0.629 | 0.627 |
| Leptotrichia hofstadii | PA(17:1/22:4) | Immobility time | 0.03 | -0.38 | 0.231 | 0.643 | 0.572 |
| Prevotella dentalis | PG(12:0/17:1) | OT % | 0.013 | -0.164 | 0.192 | 0.643 | 0.61 |
| Prevotella dentalis | LPE 18:0 | Immobility time | 0.037 | -0.332 | 0.587 | 0.644 | 0.662 |
| Prevotella dentalis | LPE 18:0 | Total distance | -0.027 | -0.55 | 0.438 | 0.649 | 0.621 |
| Leptotrichia hofstadii | PA(17:1/22:4) | OT % | -0.011 | -0.104 | 0.213 | 0.649 | 0.553 |
| Leptotrichia hofstadii | PG(12:0/17:1) | OE % | 0.1 | -0.456 | 0.372 | 0.656 | 0.667 |
| Coprococcus eutactus | PA(17:1/22:4) | Immobility time | 0.019 | -0.929 | 0.08 | 0.657 | 0.605 |
| Prevotella dentalis | LPE 18:0 | OE % | -0.017 | -0.302 | 0.228 | 0.662 | 0.597 |
| Carnobacterium divergens | PG(12:0/17:1) | Immobility time | -0.025 | -0.285 | 0.482 | 0.666 | 0.515 |
| Coprococcus eutactus | PA(17:1/22:4) | OT % | -0.012 | -0.132 | 0.724 | 0.667 | 0.536 |
| Prevotella dentalis | LPE 18:0 | OT % | -0.035 | -0.357 | 0.181 | 0.672 | 0.694 |
| Roseburia intestinalis | PA(17:1/22:4) | OT % | -0.058 | -0.549 | 0.405 | 0.681 | 0.587 |
| Coprococcus eutactus | PG(12:0/17:1) | OE % | 0.091 | -0.84 | 0.491 | 0.684 | 0.565 |
| Bacteroides thetaiotaomicron | PG(12:0/17:1) | OT % | 0.036 | -0.376 | 0.485 | 0.685 | 0.698 |
| Sphingobacterium spiritivorum | PG(12:0/17:1) | OT % | -0.005 | -0.173 | 0.197 | 0.692 | 0.594 |
| Serratia fonticola | PG(12:0/17:1) | OE % | -0.143 | -0.805 | 0.879 | 0.694 | 0.68 |
| Terrisporobacter glycolicus | PG(12:0/17:1) | OE % | 0.019 | -0.242 | 0.324 | 0.71 | 0.611 |
| Butyrivibrio crossotus | LPE 18:0 | Total distance | -0.026 | -0.73 | 0.373 | 0.712 | 0.578 |
| Bacteroides thetaiotaomicron | PG(12:0/17:1) | Immobility time | -0.07 | -1.177 | 0.569 | 0.713 | 0.673 |
| Carnobacterium divergens | PA(17:1/22:4) | Immobility time | -0.015 | -0.246 | 0.414 | 0.716 | 0.575 |
| Butyrivibrio crossotus | PG(12:0/17:1) | OT % | 0.011 | -0.236 | 0.248 | 0.72 | 0.672 |
| Carnobacterium divergens | PG(12:0/17:1) | OE % | -0.032 | -0.285 | 0.554 | 0.723 | 0.532 |
| Bacteroides thetaiotaomicron | PA(17:1/22:4) | Immobility time | -0.054 | -0.846 | 0.601 | 0.729 | 0.604 |
| Clostridium sp. SY8519 | PA(17:1/22:4) | OE % | -0.047 | -0.414 | 0.568 | 0.729 | 0.791 |
| Clostridium sp. SY8519 | PA(17:1/22:4) | OT % | 0.016 | -0.142 | 0.599 | 0.73 | 0.743 |
| Roseburia hominis | PG(12:0/17:1) | OE % | 0.053 | -0.659 | 0.585 | 0.731 | 0.528 |
| Terrisporobacter glycolicus | LPE 18:0 | Total distance | -0.014 | -0.224 | 0.303 | 0.738 | 0.563 |
| Terrisporobacter glycolicus | LPE 18:0 | Immobility time | 0.019 | -0.248 | 0.204 | 0.74 | 0.633 |
| Carnobacterium divergens | LPE 18:0 | OE % | -0.028 | -0.267 | 0.268 | 0.741 | 0.594 |
| Leptotrichia hofstadii | PG(12:0/17:1) | Total distance | -0.035 | -0.329 | 0.969 | 0.743 | 0.508 |
| Terrisporobacter glycolicus | PA(17:1/22:4) | OE % | 0.016 | -0.15 | 0.377 | 0.747 | 0.663 |
| Bacteroides thetaiotaomicron | LPE 18:0 | Total distance | -0.01 | -0.455 | 0.216 | 0.753 | 0.62 |
| Butyrivibrio crossotus | LPE 18:0 | Immobility time | 0.034 | -0.418 | 0.742 | 0.753 | 0.606 |
| Terrisporobacter glycolicus | PA(17:1/22:4) | OT % | -0.003 | -0.057 | 0.156 | 0.764 | 0.596 |
| Terrisporobacter glycolicus | LPE 18:0 | OE % | -0.006 | -0.187 | 0.191 | 0.765 | 0.598 |
| Leptotrichia hofstadii | PA(17:1/22:4) | Total distance | -0.015 | -0.304 | 0.614 | 0.773 | 0.526 |
| Butyrivibrio crossotus | LPE 18:0 | OT % | -0.028 | -0.39 | 0.354 | 0.777 | 0.58 |
| Butyrivibrio crossotus | LPE 18:0 | OE % | -0.009 | -0.239 | 0.459 | 0.785 | 0.581 |
| Bacteroides thetaiotaomicron | LPE 18:0 | Immobility time | 0.014 | -0.215 | 0.453 | 0.793 | 0.597 |
| Bacteroides thetaiotaomicron | PG(12:0/17:1) | OE % | -0.108 | -1.311 | 0.833 | 0.794 | 0.649 |
| Roseburia intestinalis | PA(17:1/22:4) | Total distance | -0.106 | -1.778 | 0.861 | 0.795 | 0.525 |
| Bacteroides thetaiotaomicron | LPE 18:0 | OE % | -0.006 | -0.181 | 0.231 | 0.804 | 0.525 |
| Terrisporobacter glycolicus | LPE 18:0 | OT % | -0.016 | -0.155 | 0.195 | 0.806 | 0.609 |
| Prevotella dentalis | PG(12:0/17:1) | OE % | -0.019 | -0.411 | 0.392 | 0.809 | 0.523 |
| Bacteroides thetaiotaomicron | PG(12:0/17:1) | Total distance | 0.056 | -0.804 | 1.112 | 0.813 | 0.576 |
| Butyrivibrio crossotus | PG(12:0/17:1) | OE % | -0.011 | -0.463 | 0.202 | 0.813 | 0.787 |
| Bacteroides thetaiotaomicron | PA(17:1/22:4) | Total distance | 0.046 | -0.867 | 0.839 | 0.82 | 0.558 |
| Coprococcus eutactus | PG(12:0/17:1) | Total distance | -0.02 | -0.29 | 1.188 | 0.829 | 0.621 |
| Bacteroides thetaiotaomicron | PA(17:1/22:4) | OT % | 0.013 | -0.365 | 0.267 | 0.835 | 0.517 |
| Bacteroides thetaiotaomicron | LPE 18:0 | OT % | -0.013 | -0.311 | 0.138 | 0.837 | 0.6 |
| Sphingobacterium spiritivorum | PG(12:0/17:1) | OE % | 0.007 | -0.141 | 0.376 | 0.838 | 0.697 |
| Sphingobacterium spiritivorum | PA(17:1/22:4) | Immobility time | 0.004 | -0.285 | 0.334 | 0.844 | 0.637 |
| Terrisporobacter glycolicus | PG(12:0/17:1) | Total distance | 0.005 | -0.167 | 0.34 | 0.855 | 0.687 |
| Prevotella dentalis | PG(12:0/17:1) | Immobility time | -0.004 | -0.459 | 0.419 | 0.857 | 0.523 |
| Prevotella dentalis | PA(17:1/22:4) | OT % | -0.001 | -0.225 | 0.173 | 0.858 | 0.557 |
| Coprococcus eutactus | PA(17:1/22:4) | Total distance | -0.007 | -0.126 | 1.151 | 0.863 | 0.677 |
| Roseburia intestinalis | PG(12:0/17:1) | Total distance | -0.109 | -1.83 | 1.071 | 0.876 | 0.512 |
| Clostridium sp. SY8519 | PA(17:1/22:4) | Total distance | -0.01 | -0.362 | 1.514 | 0.877 | 0.62 |
| Roseburia intestinalis | PG(12:0/17:1) | Immobility time | -0.079 | -1.251 | 1.007 | 0.882 | 0.603 |
| Prevotella dentalis | PA(17:1/22:4) | OE % | 0.006 | -0.276 | 0.291 | 0.886 | 0.524 |
| Butyrivibrio crossotus | PA(17:1/22:4) | Immobility time | -0.008 | -0.567 | 0.597 | 0.894 | 0.656 |
| Butyrivibrio crossotus | PG(12:0/17:1) | Total distance | -0.004 | -0.377 | 0.824 | 0.898 | 0.608 |
| Roseburia intestinalis | PA(17:1/22:4) | Immobility time | -0.039 | -1.038 | 1.073 | 0.899 | 0.585 |
| Butyrivibrio crossotus | PA(17:1/22:4) | Total distance | -0.008 | -0.978 | 0.601 | 0.904 | 0.562 |
| Sphingobacterium spiritivorum | PG(12:0/17:1) | Total distance | 0.002 | -0.42 | 0.445 | 0.907 | 0.589 |
| Roseburia hominis | PG(12:0/17:1) | Immobility time | 0.004 | -1.049 | 0.286 | 0.918 | 0.675 |
| Roseburia hominis | PA(17:1/22:4) | OT % | 0.001 | -0.072 | 0.533 | 0.919 | 0.646 |
| Sphingobacterium spiritivorum | PG(12:0/17:1) | Immobility time | 0.001 | -0.347 | 0.373 | 0.929 | 0.607 |
| Roseburia hominis | PA(17:1/22:4) | OE % | -0.005 | -0.364 | 0.456 | 0.931 | 0.51 |
| Terrisporobacter glycolicus | PA(17:1/22:4) | Immobility time | 0.001 | -0.34 | 0.2 | 0.935 | 0.562 |
| Carnobacterium divergens | PG(12:0/17:1) | Total distance | -0.004 | -0.863 | 0.366 | 0.936 | 0.632 |
| Terrisporobacter glycolicus | PA(17:1/22:4) | Total distance | 0.001 | -0.209 | 0.403 | 0.942 | 0.57 |
| Sphingobacterium spiritivorum | PA(17:1/22:4) | Total distance | 0.001 | -0.304 | 0.373 | 0.949 | 0.574 |
| Sphingobacterium spiritivorum | LPE 18:0 | Total distance | 0.001 | -0.366 | 0.24 | 0.957 | 0.525 |
| Butyrivibrio crossotus | PG(12:0/17:1) | Immobility time | -0.001 | -0.682 | 0.226 | 0.957 | 0.663 |
| Roseburia hominis | PG(12:0/17:1) | Total distance | -0.001 | -0.245 | 1.592 | 0.959 | 0.691 |
| Clostridium sp. SY8519 | PG(12:0/17:1) | OT % | 0.001 | -0.787 | 0.509 | 0.962 | 0.588 |
| Terrisporobacter glycolicus | PG(12:0/17:1) | Immobility time | 0.001 | -0.263 | 0.166 | 0.965 | 0.617 |
| Prevotella dentalis | PG(12:0/17:1) | Total distance | -0.001 | -0.455 | 0.56 | 0.966 | 0.556 |
| Sphingobacterium spiritivorum | LPE 18:0 | Immobility time | -0.002 | -0.284 | 0.398 | 0.966 | 0.508 |
| Sphingobacterium spiritivorum | LPE 18:0 | OE % | 0.001 | -0.199 | 0.19 | 0.966 | 0.552 |
| Roseburia hominis | LPE 18:0 | Total distance | 0.003 | -0.627 | 1.657 | 0.97 | 0.527 |
| Roseburia hominis | LPE 18:0 | Immobility time | -0.003 | -2.026 | 0.738 | 0.971 | 0.512 |
| Prevotella dentalis | PA(17:1/22:4) | Total distance | 0 | -0.363 | 0.911 | 0.972 | 0.565 |
| Prevotella dentalis | PA(17:1/22:4) | Immobility time | 0 | -0.647 | 0.434 | 0.974 | 0.534 |
| Roseburia hominis | PA(17:1/22:4) | Immobility time | 0 | -1.056 | 0.24 | 0.975 | 0.604 |
| Sphingobacterium spiritivorum | LPE 18:0 | OT % | 0.001 | -0.289 | 0.193 | 0.978 | 0.522 |
| Clostridium sp. SY8519 | PG(12:0/17:1) | OE % | -0.001 | -1.459 | 0.029 | 0.979 | 0.858 |
| Clostridium sp. SY8519 | PG(12:0/17:1) | Total distance | 0 | -0.408 | 2.199 | 0.983 | 0.682 |
| Roseburia hominis | PA(17:1/22:4) | Total distance | 0 | -0.244 | 1.215 | 0.983 | 0.642 |
| Clostridium sp. SY8519 | PA(17:1/22:4) | Immobility time | 0.001 | -1.278 | 0.517 | 0.983 | 0.649 |
| Roseburia hominis | LPE 18:0 | OE % | 0.001 | -0.273 | 1.76 | 0.983 | 0.555 |
| Roseburia intestinalis | LPE 18:0 | Total distance | 0.001 | -0.398 | 0.877 | 0.984 | 0.532 |
| Roseburia intestinalis | LPE 18:0 | Immobility time | -0.001 | -0.858 | 0.471 | 0.992 | 0.523 |
| Clostridium sp. SY8519 | PG(12:0/17:1) | Immobility time | 0 | -1.86 | 0.461 | 0.994 | 0.679 |
| Roseburia intestinalis | LPE 18:0 | OE % | 0 | -0.672 | 0.282 | 0.994 | 0.612 |
| Roseburia hominis | LPE 18:0 | OT % | 0.003 | -0.423 | 1.184 | 0.994 | 0.51 |
| Carnobacterium divergens | PA(17:1/22:4) | Total distance | 0 | -0.474 | 0.344 | 0.995 | 0.62 |
| Roseburia intestinalis | LPE 18:0 | OT % | 0.001 | -0.392 | 0.48 | 0.998 | 0.504 |

**Supplementary table 6**. Mediation effect parameters and stability estimates for the microbiota-metabolite-behavior axis in the [(HFD-CUMS vs. ND-CUMS) ∩ (HFD-CUMS vs. CTL)] group.

| Microbiota | Metabolites | Phenotype | ACME | 95% CI  (Lower) | 95% CI  (Upper) | P-value  (Bootstrap) | Stability Rate |
| --- | --- | --- | --- | --- | --- | --- | --- |
| Fibrella aestuarina | Cer-BS d27:2 | Total distance | -0.154 | -0.586 | 0.158 | 0.508 | 0.878 |
| Sphingobacterium thalpophilum | Cer-BS d27:2 | Total distance | 0.109 | -0.137 | 0.697 | 0.547 | 0.855 |
| Zunongwangia profunda | Cer-BS d27:2 | Total distance | -0.088 | -0.433 | 0.205 | 0.593 | 0.779 |
| Fibrella aestuarina | Cer-BS d27:2 | OT % | 0.015 | -0.081 | 0.167 | 0.677 | 0.646 |
| Fibrella aestuarina | Cer-BS d27:2 | OE % | 0.017 | -0.155 | 0.237 | 0.691 | 0.708 |
| Zunongwangia profunda | Cer-BS d27:2 | Immobility time | -0.014 | -0.198 | 0.279 | 0.705 | 0.576 |
| Bifidobacterium choerinum | Cer-BS d27:2 | Immobility time | -0.008 | -0.271 | 0.288 | 0.718 | 0.524 |
| Bifidobacterium choerinum | Cer-BS d27:2 | Total distance | -0.034 | -0.273 | 0.378 | 0.730 | 0.609 |
| Bifidobacterium animalis | Cer-BS d27:2 | Total distance | -0.034 | -0.381 | 0.290 | 0.747 | 0.537 |
| Paenibacillus sonchi | Cer-BS d27:2 | Immobility time | -0.007 | -0.412 | 0.422 | 0.776 | 0.520 |
| Paenibacillus sonchi | Cer-BS d27:2 | Total distance | -0.036 | -0.514 | 0.568 | 0.793 | 0.562 |
| Sphingobacterium thalpophilum | Cer-BS d27:2 | OE % | -0.005 | -0.710 | 0.227 | 0.795 | 0.581 |
| Sphingobacterium thalpophilum | Cer-BS d27:2 | Immobility time | 0.006 | -0.390 | 0.497 | 0.805 | 0.528 |
| Paenibacillus sonchi | Cer-BS d27:2 | OT % | -0.003 | -0.393 | 0.172 | 0.812 | 0.527 |
| Bifidobacterium animalis | Cer-BS d27:2 | OT % | -0.002 | -0.119 | 0.116 | 0.838 | 0.525 |
| Bifidobacterium animalis | Cer-BS d27:2 | Immobility time | -0.004 | -0.155 | 0.164 | 0.841 | 0.509 |
| Bifidobacterium choerinum | Cer-BS d27:2 | OT % | -0.001 | -0.150 | 0.132 | 0.865 | 0.642 |
| Bifidobacterium choerinum | Cer-BS d27:2 | OE % | 0.002 | -0.212 | 0.198 | 0.877 | 0.525 |
| Paenibacillus sonchi | Cer-BS d27:2 | OE % | 0.001 | -0.514 | 0.313 | 0.935 | 0.574 |
| Bifidobacterium animalis | Cer-BS d27:2 | OE % | 0.000 | -0.166 | 0.175 | 0.973 | 0.533 |
| Fibrella aestuarina | Cer-BS d27:2 | Immobility time | -0.001 | -0.177 | 0.230 | 0.980 | 0.515 |
| Sphingobacterium thalpophilum | Cer-BS d27:2 | OT % | 0.000 | -0.371 | 0.145 | 0.985 | 0.504 |
| Zunongwangia profunda | Cer-BS d27:2 | OE % | 0.000 | -0.161 | 0.266 | 0.990 | 0.549 |
| Zunongwangia profunda | Cer-BS d27:2 | OT % | 0.000 | -0.113 | 0.103 | 0.998 | 0.533 |
